# Haplotype-tagged SNPs improve genomic prediction accuracy for Fusarium head blight resistance and yield-related traits in wheat

**DOI:** 10.1007/s00122-023-04352-8

**Published:** 2023-04-03

**Authors:** Admas Alemu, Lorena Batista, Pawan K. Singh, Alf Ceplitis, Aakash Chawade

**Affiliations:** 1 Department of Plant Breeding, Swedish University of Agricultural Sciences, Alnarp, Sweden; 2 Lantmännen Lantbruk, Svalöv, Sweden; 3 International Maize and Wheat Improvement Center, Texcoco, Mexico

## Abstract

**Key message:**

Linkage disequilibrium (LD)-based haplotyping with subsequent SNP tagging improved the genomic prediction accuracy up to 0.07 and 0.092 for Fusarium head blight resistance and spike width, respectively, across six different models.

**Abstract:**

Genomic prediction is a powerful tool to enhance genetic gain in plant breeding. However, the method is accompanied by various complications leading to low prediction accuracy. One of the major challenges arises from the complex dimensionality of marker data. To overcome this issue, we applied two pre-selection methods for SNP markers viz. LD-based haplotype-tagging and GWAS-based trait-linked marker identification. Six different models were tested with preselected SNPs to predict the genomic estimated breeding values (GEBVs) of four traits measured in 419 winter wheat genotypes. Ten different sets of haplotype-tagged SNPs were selected by adjusting the level of LD thresholds. In addition, various sets of trait-linked SNPs were identified with different scenarios from the training-test combined and only from the training populations. The BRR and RR-BLUP models developed from haplotype-tagged SNPs had a higher prediction accuracy for FHB and SPW by 0.07 and 0.092, respectively, compared to the corresponding models developed without marker pre-selection. The highest prediction accuracy for SPW and FHB was achieved with tagged SNPs pruned at weak LD thresholds (*r*^2^ < 0.5), while stringent LD was required for spike length (SPL) and flag leaf area (FLA). Trait-linked SNPs identified only from training populations failed to improve the prediction accuracy of the four studied traits. Pre-selection of SNPs via LD-based haplotype-tagging could play a vital role in optimizing genomic selection and reducing genotyping costs. Furthermore, the method could pave the way for developing low-cost genotyping methods through customized genotyping platforms targeting key SNP markers tagged to essential haplotype blocks.

**Supplementary Information:**

The online version contains supplementary material available at 10.1007/s00122-023-04352-8.

## Background

Increased accessibility and cost-effectiveness of high throughput genomic data of various crops has revolutionized plant breeding shifting from phenotypic to genomic-based selection. In the last four decades, the development of several DNA marker genotyping systems has allowed using a higher number of molecular markers in genotyping platforms (Crossa et al. 2017). Genomic-assisted breeding soon became a feasible approach through characterization of allelic variations underlying important agronomic traits of several crops and their efficient integration in the germplasm improvement and varietal development processes (Varshney et al. 2021).

Linkage-based QTL mapping has been playing a huge role to identify QTL with large effect and positional cloning of associated functional genes, which are instrumental for marker-assisted selection in crop improvement (Bernardo 2020; Gupta et al. 2013; Röder et al. 1998; Su et al. 2018; Uga et al. 2013). Genome-wide association study (GWAS) has lately established overcoming the mapping resolution and other limitations of the linkage mapping and extensively used in several crops to identify QTL or quantitative trait nucleotides (QTNs) (Alemu et al. 2021b, 2022; Challa and Neelapu 2018; He et al. 2021; Tibbs Cortes et al. 2021; Yano et al. 2016). However, the sheer amount of identified QTL with minor to major effect hindered the immediate implementation of GWAS results for cultivar improvement. For instance, considering 30 unlinked QTL, the chance of a recombinant inbred accumulating the favorable allele of all QTL would be 1 in 1.07 billion (Bernardo 2016).

Genomic selection (GS) has emerged as a powerful genomic-assisted breeding method overcoming several of the limitations in QTL mapping approaches. Genomic selection was proposed nearly two decades ago (Meuwissen et al. 2001) but has been routinely applied in plant breeding only recently enabled by the reduced costs of high-throughput genotyping technologies. Genomic selection, over phenotypic selection, reduces both the cost per cycle and required time/breeding cycle (rapid selection cycle) and improves the development of crop varieties in several breeding programs (Crossa et al. 2017; Heffner et al. 2009). Genomic prediction estimates the breeding values of individual genotypes based on their overall molecular marker information or genetic merits. Therefore, unlike QTL mapping methods, the goal of genomic prediction is to predict the breeding or genetic values of candidate genotypes. In genomic prediction, a regression model is trained with molecular markers and phenotypic data from a population called training/calibration and used to predict the genomic estimated breeding values (GEBV) of individuals in the breeding/validation/test set having only molecular marker information (Hastie et al. 2009). Then, selection of candidate individuals in the breeding population could be done solely based on their predicted genetic merit without the need to test on field for phenotypic evaluation.

For the last couple of decades, several statistical methods have been proposed for genomic prediction in both animal and plant breeding programs such as GBLUP, RR-BLUP, BayesA, BayesB, BayesC, Bayesian LASSO (BL), Bayesian Ridge Regression (BRR) and Reproducing Kernel Hilbert Space (RKHS) (De Los Campos et al. 2009; Endelman 2011; Meuwissen et al. 2001; Pérez and De Los Campos 2014). Several factors contribute to the performance of genomic prediction models in plant breeding, such as the genetic architecture of a trait, heritability, sample size and diversity of the training population, genetic relatedness between the training and breeding population, span and extent of linkage disequilibrium between markers and QTL and distribution of SNP markers (Crossa et al. 2017; Pérez and De Los Campos 2014).

In genomic prediction, the number of predictors (i.e., SNP markers) (*p*) is generally much larger than the number of observations (*n*) (*p* >  > *n*). Genome-wide markers covering the entire genome are critical in this method to attest inclusion of all major- and minor-effect QTL of a target trait (Desta and Ortiz 2014). However, the presence of several markers with no effect increases noise in genomic prediction models and reduces the accuracy as evidenced in previous studies (Meher et al. 2022; Pang et al. 2021; Schulz-Streeck et al. 2011). Besides, studies have shown that increasing marker density could negatively affect the prediction accuracy of Bayesian-based models due to the slow or non-convergence of the Markov Chain Monte Carlo (MCMC) iterations (Zhang et al. 2019). The other major challenge arises from the high dimensionality and multicollinearity of marker data used to develop genomic prediction models. Adjacent markers tend to have a high correlation that leads to multicollinearity in the prediction models (Crossa et al. 2017; Neves et al. 2012; Wang et al. 2015). Statistical models such as partial least square (PLS) (Boulesteix and Strimmer 2006), principal components regression (PCR) (Du et al. 2018) and sparse partial least square regression (SPLS) (Chung and Keles 2010) proposed to reduce the high dimensionality and multicollinearity of SNP markers in genomic prediction. However, these models have not been widely used as various studies have shown a similar or low prediction accuracy compared to the commonly used BLUP- or Bayesian-based models (Lorenz et al. 2011; Thavamanikumar et al. 2015; Xu et al. 2017).

Haplotyping of SNP markers and fitting haplotype blocks in prediction models instead of single SNPs could alleviate the different shortcomings mentioned previously. A haplotype is a set of SNP markers, or other genomic structural variants, found nearby on a particular chromosome and is under linkage disequilibrium (Bhat et al. 2021; Nordborg and Tavaré 2002; Qian et al. 2017). These alleles of various polymorphisms existing on a segment of chromosome are inherited together with a minimum chance of contemporary recombination (Garg 2021; Sehgal et al. 2020). The majority of genomic selection models in plant breeding programs account for all SNP markers generated from high throughput genotyping technologies. However, haplotype-based genomic prediction has proven an efficient method to improve the prediction accuracy in animal breeding programs (Cuyabano et al. 2014; Li et al. 2021; Won et al. 2020). The use of haplotypes could improve genomic prediction accuracy because it can capture the LD between markers and QTL more efficiently, make the genomic similarity in different lines more clear and also capture local high-order allelic interactions or local epistatic effects (Bhat et al. 2021; Habier et al. 2007; He et al. 2019; Jiang et al. 2018).

A multi-allelic haplotype-based genomic prediction study has shown its potential to improve the genomic prediction accuracy of different traits in wheat (Sallam et al. 2020). However, the method is based on grouping of SNP markers to particular haplotype blocks creating haplotype alleles that does not overcome the problems of over-fitting and other biases caused by the higher numbers of markers. To overcome this problem, the current study developed genomic prediction models based on SNP markers tagged to haplotype blocks selected by fine-tuning to 10 different LD thresholds and tested the accuracy in five Bayesian-based and RR-BLUP models. For this purpose, 419 winter wheat genotypes comprising two separate populations were exploited with different cross-validation scenarios to estimate the genomic breeding values of Fusarium head blight (FHB), spike length (SPL), spike width (SPW) and flag leaf area (FLA). Identification of various sets of SNPs linked to a particular trait via GWAS was the other marker pre-selection method applied on the current genomic prediction models. Finally, the prediction accuracy within and between populations was compared for models using haplotype-tagged, trait-linked, and all SNP markers.

## Materials and methods

### Plant material and phenotypic data

The current study utilized 419 winter wheat genotypes, including 272 advanced breeding lines developed by the breeding company Lantmännen Lantbruk, Svalöv, Sweden and 147 old cultivars and landraces (genebank lines hereafter) preserved in the Nordic Genetic Resource Center (NordGen) gene bank, Alnarp, Sweden. The collection was previously evaluated by Zakieh et al. (2021) for Fusarium head blight resistance and other yield-related traits (i.e., spike length, spike width and flag leaf area) under accelerated growth conditions in a controlled environment with subsequent QTL identification. The current genomic prediction analysis used the adjusted mean of the phenotypic data from this experiment recorded for the four traits. The details on the experimental design and phenotypic data analysis can be found in Zakieh et al. (2021). Briefly, genotypes were tested in an augmented design replicated four times. The four well known winter wheat cultivars Nimbus, Stigg, Norin, and Julius were included within each block as checks making a total of 11 and 6 blocks/replicate for breeding and genebank lines, respectively.

### Genotypic data

The genebank lines were genotyped for SNP markers with a 20 K SNP assay as described by Odilbekov et al. (2019) followed by quality checks by Alemu et al. (2021a). The breeding lines were genotyped with 25 K SNP assay as described by Zakieh et al. (2021) and applied the same quality checking standards as for genebank lines. After the quality checking, 6421 SNP markers common to both populations were identified and applied for genomic prediction model development.

### Genomic prediction models

Six different genomic prediction models were tested with various sets of SNP markers including SNPs preselected through GWAS, haplotype-tagged SNPs with ten selected LD thresholds and with non-preselected SNPs making 15 different scenarios (Table 1). The *rrBLUP* package (Endelman 2011) in R environment (R Core Team 2022) was used to develop the RR-BLUP model fitting the basic linear mixed model:

$$Y = 1\beta + Z\mu + \varepsilon$$

where *Y* is the *N* × 1 vector of adjusted phenotypic means (BLUPs) of each of the four tested traits (i.e., FHB, SPL, SPW, FLA); *β* is the intercept; *Z* is the *N* × Nm SNP markers matrix developed from either haplotype-tagged, trait-linked or non-preselected SNPs; *N* and Nm stand for number of genotypes and SNP markers, respectively; *μ* is the Nm × 1 vector of random SNP effects obtained in the “*mixed.solve*” function following *μ*∼*N* (0, I${\sigma }_{m}^{2}$) where ${\sigma }_{m}^{2}$ is the genetic variance component contributed in each SNP marker and I is the identity matrix; and ε is the *N* × 1 vector of residual effects.

**Table 1:** The various genomic prediction analysis scenarios applied with marker pre-selection methods and training-test combinations

Scenarios	Cross-validation	Training set (TRS)	Test set (TS)	SNP pre-selection method	Selected SNPs	Genomic prediction models	Cross-validation reps
1	Combined population: 80/20 split	BL and GL (80%)	BL and GL (20%)	Haplotyping	Haplotype-tagged	RR-BLUP, BL, BRR, BayesA, BayesB, BayesC	100 for all models except RR-BLUP & 500 for RR-BLUP
2	Combined population: 80/20 split	BL and GL (80%)	BL and GL (20%)	GWAS with TRS-TS combined	Trait-linked	RR-BLUP	500
3	Within population: 80/20 split	BL (80%)	BL (20%)	Haplotyping	Haplotype-tagged	RR-BLUP, BL, BRR, BayesA, BayesB, BayesC	100 for all models except RR-BLUP & 500 for RR-BLUP
4	Within population: fivefold	BL (4 folds)	BL (onefold)	GWAS with TS	Trait-linked	RR-BLUP	5
5	Within population: fivefold	BL (4 folds)	BL (onefold)	GWAS with TRS-TS combined	Trait-linked	RR-BLUP	5
6	Between populations	BL	GL	Haplotyping	Haplotype-tagged	RR-BLUP	–
7	Between populations	GL	BL	Haplotyping	Haplotype-tagged	RR-BLUP	–
8	Between populations	BL	GL	GWAS with TRS-TS combined	Trait-linked	RR-BLUP	–
9	Between populations	GL	BL	GWAS with TRS-TS combined	Trait-linked	RR-BLUP	–
10	Between populations	BL	GL	GWAS with TS	Trait-linked	RR-BLUP	–
11	Between populations	GL	BL	GWAS with TS	Trait-linked	RR-BLUP	–
12	Combined population: 80/20 split	BL and GL (80%)	BL and GL (20%)	–	–	RR-BLUP, BL, BRR, BayesA, BayesB, BayesC	100 for all models except RR-BLUP & 500 for RR-BLUP
13	Within population: fivefold	BL (4 folds)	BL (onefold)	–	–	RR-BLUP, BL, BRR, BayesA, BayesB, BayesC	5
14	Between populations	BL	GL	–	–	RR-BLUP	–
15	Between populations	GL	BL	–	–	RR-BLUP	–

*BL* Breeding lines; *GL* Genebank lines; *TRS* Training set; *TS* Test set; *RR-BLUP* Ridge-regression best linear unbiased prediction; *BL* Bayesian LASSO; *BRR* Bayesian ridge regression

Five different Bayesian-based models available in the *BGLR* package (Pérez and De Los Campos 2014) were tested with the same sets of SNP markers mentioned above. The majority of Bayesian models parameterize the effect of markers differently and assume non-equal genetic variance for markers effect across chromosomes in order to consider QTL with major effects. The different prior assumptions implemented in these models play an essential role in defining the type of shrinkage or variable selection imposed on the estimates of effects induced (Pérez and De Los Campos 2014). The BRR model follows a Gaussian prior that shrunk markers effect with similar extent. The scaled-t density prior used in BayesA (Meuwissen et al. 2001) and double exponential densities or Laplace prior used in the BL models are with higher mass at zero and thicker tails than the normal density that induces a size-of-effect dependent shrinkage estimates (Gianola 2013). The BayesC and BayesB models implement two finite mixture priors: the earlier use a mixture of a point of mass at zero and a Gaussian slab (Habier et al. 2011), and the later implements a mixture of a point of mass at zero and a scaled-t slab (Meuwissen et al. 2001). All *BGLR* analyses were computed with Markov Chains Monte Carlo sampler with chain length of 12,000 iterations and 10 thinning interval with the first 2000 used as burn-in.

### Selection of SNP markers for genomic prediction

The SNP markers were preselected with two approaches: I) Haplotyping by fine-tuning to 10 selected linkage disequilibrium thresholds with subsequent SNP tagging for each haplotype block and II) Various sets of GWAS-identified SNP markers significantly linked to a particular trait (Table 1).

### Haplotyping and SNP-tagging

Haplotyping of SNP markers was done separately for each of the 21 chromosomes using Haploview (Barrett et al. 2005) with default parameters but fine-tuning the extent of LD to ten selected thresholds. The chromosome-wide LD was measured as pairwise *r*^2^ values. A haplotype “*SNP-Tagger*” function (De Bakker et al. 2005) plugged into the haploview algorithm was applied to tag and select a representative SNP marker through prioritizing tags. The method considered all alleles that can serve as a proxy at a given linkage disequilibrium extent, followed by prioritizing a SNP marker tagged per haplotype block. Ten different linkage disequilibrium threshold levels (*r*^2^ = 0.1–1.0) were selected for haplotyping and subsequent tagged SNPs selection to develop genomic prediction models. The haplotyping and SNP tagging analysis was conducted for breeding and genebank lines separately, and the common tagged markers were selected for the two populations combined genomic prediction analysis. In addition, the tagged SNPs identified only from the breeding lines were separately tested in the genomic prediction models.

### Trait-linked SNP markers

The multi-locus GWAS model, Fixed and random model Circulating Probability Unification (FarmCPU) (Liu et al. 2016) in GAPIT (Wang and Zhang 2021) was employed to identify SNP markers linked to a specific trait. FarmCPU took the advantage of both the mixed linear model and the linear regression model and overcome their disadvantages by using them iteratively (Liu et al. 2016). In FarmCPU, a special kinship matrix is created from markers associated with the causal QTL, also called pseudo-quantitative trait nucleotides, in the mixed model. The identified set of pseudo-quantitative trait nucleotides are then fitted as covariates in the linear model to test markers effect to traits of interest (Liu et al. 2016). The GWAS analysis was performed with (I) the whole set of genotypes including both the 272 breeding lines and 147 genebank lines, (II) from the two populations separately and later used to train the genomic prediction model on the other population and (III) from only 80% of the 272 breeding lines used as training population (Table 1). The third method was tested only for breeding lines since the number of genotypes in the genebank lines is inadequate to conduct GWAS from 80% of the 147 genotypes. The GWAS analysis for trait-linked SNPs selection was done from only training populations as well as from the training-test combined populations. The later was done to demonstrate the inflation of genomic prediction accuracy when the GWAS and genomic prediction analysis are non-independent due to the overfitting of the genomic prediction analysis and lead an increased prediction accuracy. Genomic prediction models were tested with only selected SNP markers linked to a particular trait separately based on their significance level.

### Cross-validation analysis

#### Within populations

The within (BL) and combined populations (BL and GL) GP accuracy was computed through cross-validation by randomly setting 80% and 20% of the genotypes as training and test sets, respectively (Table 1). Preselected markers identified from the two pooled populations along with non-preselected/all SNP markers were tested in the five Bayesian and RR-BLUP models via 80–20% cross-validation method (Scenarios 1, 2 and 12). In addition, genomic prediction accuracy of haplotype-tagged SNPs identified only from breeding lines was computed through this method (Scenarios 3 and 13). With tagged SNPs, the five different Bayesian-based and RR-BLUP models were tested with the identified sets of selected markers in 10 different LD thresholds separately. For trait-linked SNPs, markers identified from the two combined populations (Scenario 2) were first arranged based on their significance (i.e., from low to high *P*-values) in the GWAS analysis to a particular trait. Then, the RR-BLUP model was evaluated with sets of significant SNP markers located at 1–100, 101–201, 1–200, 201–300, 1–300, all except the first 300 GWAS SNPs and two randomly selected 100 SNP markers.

The cross-validation analysis was repeated for 100 and 500 times in the five Bayesian and RR-BLUP models, respectively. The predictive abilities of models were assessed from the correlation between the GEBVs of individuals in the test set and their BLUPs resulted from phenotypic data analysis. The prediction accuracy was estimated as a fraction of the predictive ability from the square root of the broad-sense heritability of the traits (Legarra et al. 2008).

#### Between populations

The independent population genomic prediction accuracy was tested among breeding and genebank lines using the RR-BLUP model. The between-populations prediction analysis was established with eight different scenarios resulted from the two populations and two SNP pre-selection methods combinations (Scenarios 6, 7, 8, 9, 10, 11, 14 and 15). Haplotype-tagged SNPs shared with the breeding and genebank lines were tested for genomic prediction across the two independent populations using the six models. Three sets of trait-linked SNP markers identified from the pooled as well as only from the training population were used to test across population genomic prediction accuracy using the RR-BLUP model (Scenarios 8, 9, 10 and 11). In all the independent population cross-validation analysis, one was used to train the model, while the other population serve as test set and the analysis was repeated the other way around. Moreover, within population genomic prediction was tested with several sets of trait-linked SNPs (200–3000) identified only from the training sets (Scenario 4) of 272 breeding lines following the five-fold cross-validation genomic prediction scheme or with GWAS-SNPs identified from the population comprising both the training and test individuals (Scenario 5).

## Results

### Comparing genomic prediction models with non-preselected SNPs

The breeding and genebank lines were pooled and tested with non-preselected or all available SNP markers via 80–20% training-test set cross-validation analysis with six different prediction models. The five Bayesian and the RR-BLUP models estimated the GEBVs of FHB and SPL with inconsiderable prediction accuracy differences. The RR-BLUP model predicted the genomic estimated breeding values of FHB and SPL slightly better than the five Bayesian models with 0.46 and 0.80 prediction accuracy, respectively (Table 2). The BayesA model predicted the GEBVs of SPW better than the other five tested models with 0.38 prediction accuracy and was improved by 0.058 compared to the RR-BLUP model. However, the RR-BLUP model predicted the GEBV of FLA with 0.59 prediction accuracy improving by 0.33 and 0.3 compared to BayesA (0.26) and BayesC (0.29), the lowest and highest scorings from Bayesian models, respectively (Table 2).

**Table 2:** Genomic prediction accuracy with non-preselected SNP markers tested in the two combined populations using six models across the four traits

Traits	RR-BLUP	BL	BRR	BayesA	BayesB	BayesC
FHB	0.462	0.459	0.433	0.452	0.448	0.441
SPL	0.796	0.795	0.786	0.783	0.783	0.793
SPW	0.321	0.336	0.358	0.379	0.370	0.330
FLA	0.593	0.272	0.267	0.259	0.265	0.285

*FHB* Fusarium head blight; *SPL* Spike length; *SPW* Spike width; *FLA* Flag leaf area**;** *RR-BLUP* Ridge-regression best linear unbiased prediction; *BL* Bayesian LASSO; *BRR* Bayesian ridge regression

### Genomic prediction with haplotype-tagged SNPs

The haplotype analysis conducted from the two populations separately identified different sets of tagged SNPs common to both populations across the ten selected LD thresholds (Table 3) and only from breeding lines (Supplementary Table S1). All the results of haplotyping and SNP tagging/haplotype blocks in the two populations can be found in Supplementary File 1. The number of identified tag-SNP markers from the combined population varied from 83 with the LD threshold at *r*^2^ = 0.1 to 6098 with *r*^2^ = 1.0, representing 1.29–94.97% of the total SNP markers, respectively. The average number of SNP markers in a haplotype block ranged from 1.05 to 77.36 identified at the applied minimum and maximum LD thresholds, respectively. A single SNP marker was identified as a haplotype across all LD thresholds, while the maximum ranged from 50 (*r*^2^ = 1.0) to 167 SNPs/haplotype block (*r*^2^ = 0.1) suggesting uneven distribution of SNPs density across chromosomes (Table 3). The number of tagged SNPs in breeding lines was ranged from 566 (*r*^2^ = 0.1) to 6098 (*r*^2^ = 1.0) (Supplementary Table S1).

**Table 3:** Haplotyping at ten selected LD thresholds, haplotype-tagged SNPs, minimum, maximum and average numbers of SNPs per haplotype identified from the genebank-breeding lines combined populations

LD thresholds (*r*^2^)^a^	Mean LD (*r*^2^)^b^	Haplotype-tagged SNPs^c^	SNPs distribution/haplotype		
			Minimum	Maximum	Average
0.1	0.50	83	1	162	77.36
0.2	0.61	270	1	101	23.78
0.3	0.71	459	1	98	13.99
0.4	0.8	690	1	96	5.12
0.5	0.84	943	1	91	9.31
0.6	0.88	1195	1	83	5.37
0.7	0.89	1542	1	72	4.16
0.8	0.95	2714	1	72	2.37
0.9	0.99	3090	1	52	2.08
1.0	1.0	6098	1	50	1.05

^a^The LD thresholds applied to select haplotype-tagged SNP markers

^b^The average LD recorded at a particular threshold

^c^The number of tagged SNP markers identified in a particular LD-threshold that subsequently used in the genomic prediction models

**Fig. 1:**
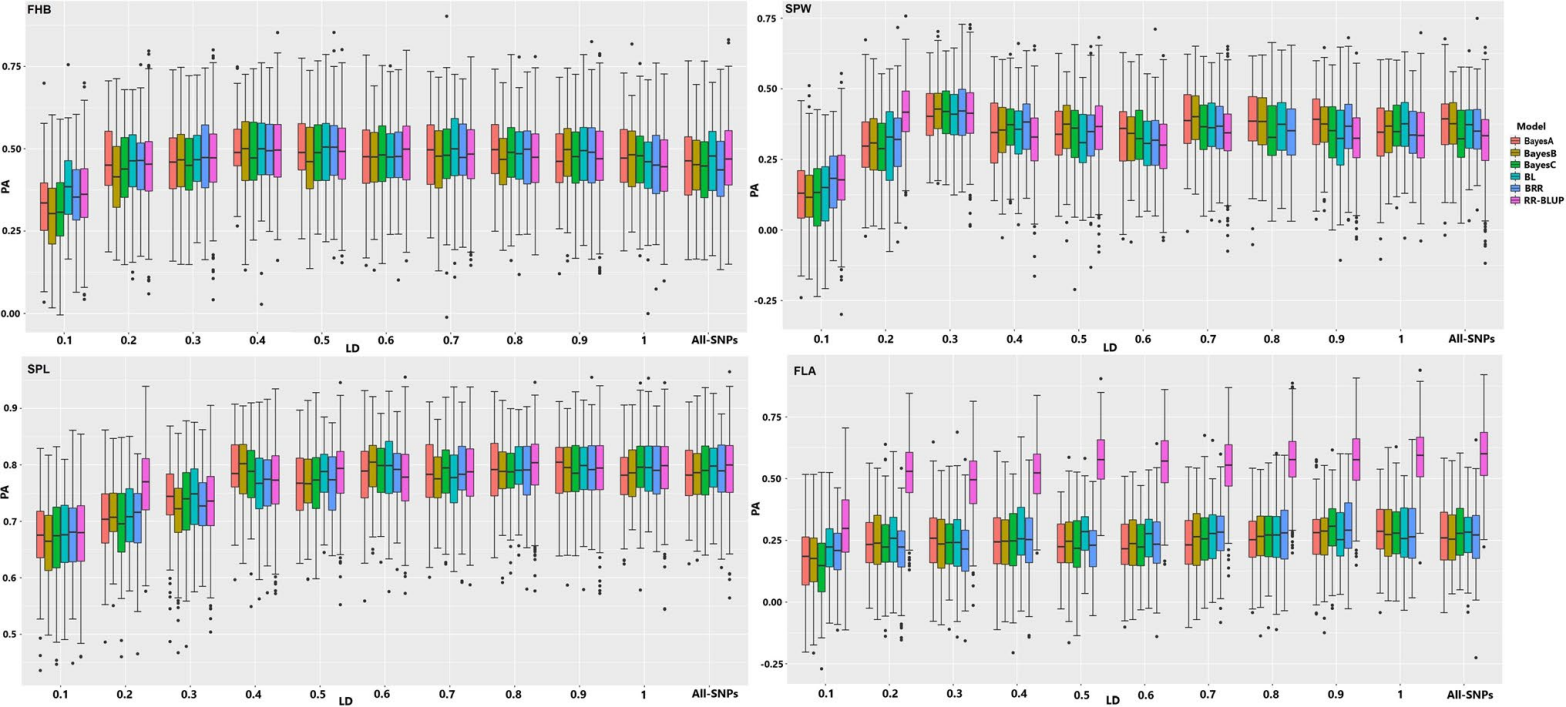
Distribution of the genomic prediction accuracy measured with haplotype-tagged SNP markers selected at ten different LD thresholds and tested in the five Bayesian and with RR-BLUP models (scenarios 1 and 12). *PA* Prediction accuracy; *LD* Linkage disequilibrium; *FHB* Fusarium head blight; *SPL* Spike length; *SPW* Spike width; *FLA* Flag leaf area

With haplotype-tagged SNPs shared with the two populations, the genomic prediction accuracy of the four traits was improved compared to the non-preselected markers in the Bayesian models (Scenario 1). In these models, tagged SNPs improved the genomic prediction accuracy up to 0.082, and except in a single case, the highest accuracy was recorded from tagged markers compared to fitting non-preselected SNPs (Fig. 1, Supplementary Table S2). The prediction accuracy improvement stretched up to 0.092 in RR-BLUP model for SPW with 459 haplotype-tagged SNPs selected at *r*^2^ = 0.3 (Fig. 1, Supplementary Table S2). Overall, tagged markers improved up to 0.012, 0.033, 0.07 and 0.092 in SPL, FLA, FHB and SPW, respectively, across all models and LD thresholds. The extent of LD threshold for optimum genomic prediction varied across models and the three traits (i.e., FHB, SPL and FLA). However, the highest genomic prediction accuracy of SPW was achieved with 459 haplotype-tagged SNPs selected at threshold of *r*^2^ = 0.3 in all the five Bayesian and RR-BLUP models (Supplementary Table S2). From the Bayesian models, BRR was the topmost to predict the GEBVs of FHB and FLA with 943 (*r*^2^ = 0.5) and 3090 (*r*^2^ = 0.9) haplotype-tagged SNPs with prediction accuracy of 0.503 and 0.3, respectively. The BRR and BayesB models with 1195 (*r*^2^ = 0.6) and 3090 (*r*^2^ = 0.9) haplotype-tagged SNPs, respectively, and BL with 6421 none-preselected SNPs predicted the GEBVs of SPL equally with highest prediction accuracy. The BayesB model was the best to predict the GEBVs of SPW using 459 (*r*^2^ = 0.3) haplotype-tagged SNP markers with 0.424 prediction accuracy. In the RR-BLUP model, the highest prediction accuracy of SPW, FHB and SPL was achieved at 459 (*r*^2^ = 0.3), 690 (*r*^2^ = 0.4) and 2714 (*r*^2^ = 0.8) haplotype-tagged SNPs with prediction accuracy of 0.413, 0.494 and 0.799, respectively. However, the none-preselected SNP markers performed better than tagged SNPs to predict for FLA with 0.593 accuracy (Supplementary Table S2).

**Fig. 2:**
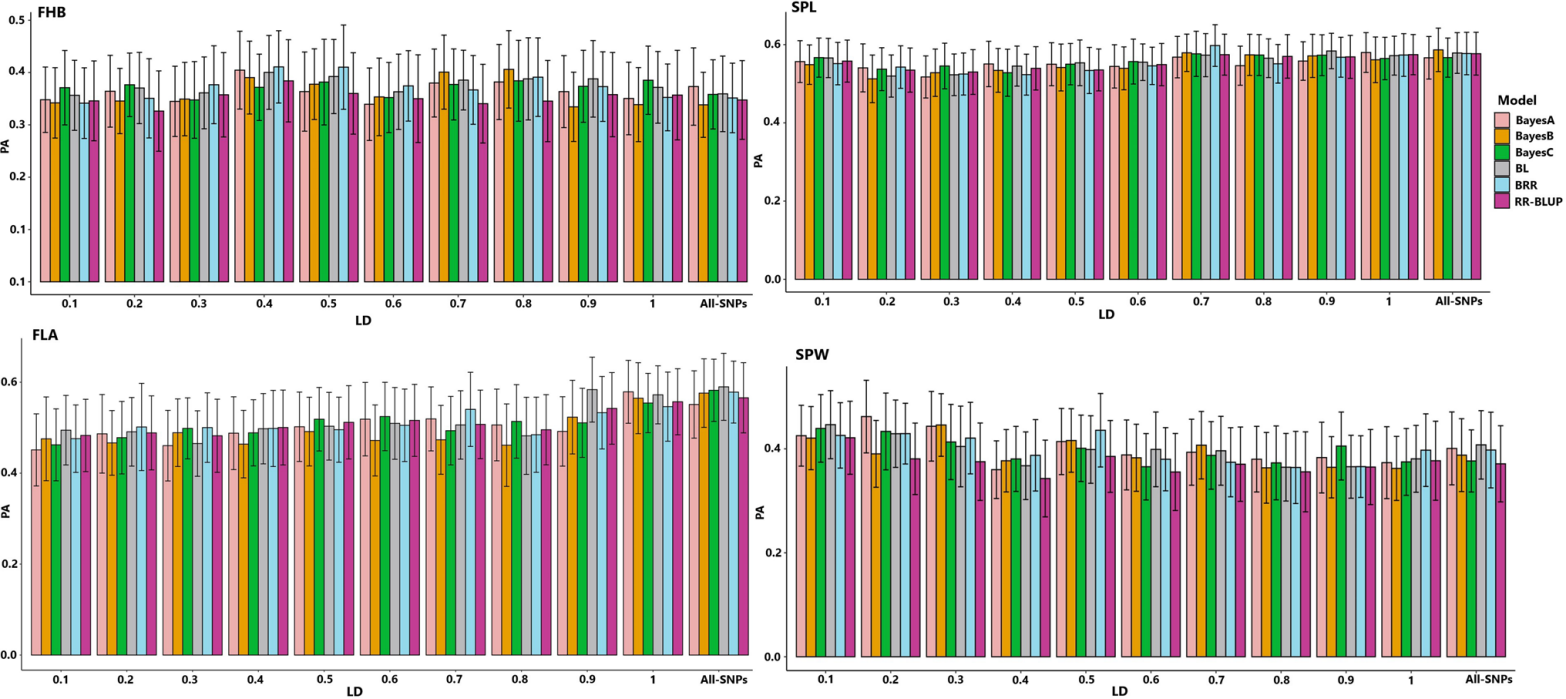
Performance of haplotype-tagged SNPs pruned at 10 selected LD thresholds in the genomic prediction of four traits with six different models tested in breeding lines (scenarios 3 and 13). *PA* Prediction accuracy; *LD* Linkage disequilibrium; *FHB* Fusarium head blight; *SPL* Spike length; *SPW* Spike width; *FLA* Flag leaf area

Using only breeding lines (Scenario 3) for model training resulted a more stable trend of prediction accuracy scores with tagged SNPs pruned at the ten LD threshold compared to the combined populations (Fig. 2). For instance, except BayeB, all the other four Bayesian models as well as the RR-BLUP model achieved the highest prediction accuracy with 1425 tagged SNPs selected at LD of *r*^2^ = 0.4 for FHB. For SPW, the four different models reached their highest prediction accuracy at LD of *r*^2^ = 0.1 with 566 tagged SNP markers. Overall, tagged SNPs selected with weak LD thresholds (*r*^2^ < 0.5) allowed models to reach their highest prediction accuracy for SPW and FHB. However, the FLA and SPL required either a stringent or non-preselected SNPs for maximum genomic prediction accuracy. Generally, the haplotype-tagged SNPs increased the prediction accuracy of SPL, FHB and SPW with 0.011, 0.044 and 0.069, respectively, but did not improve for FLA (Supplementary Table S1).

### Haplotype-based genomic prediction with independent populations

**Fig. 3:**
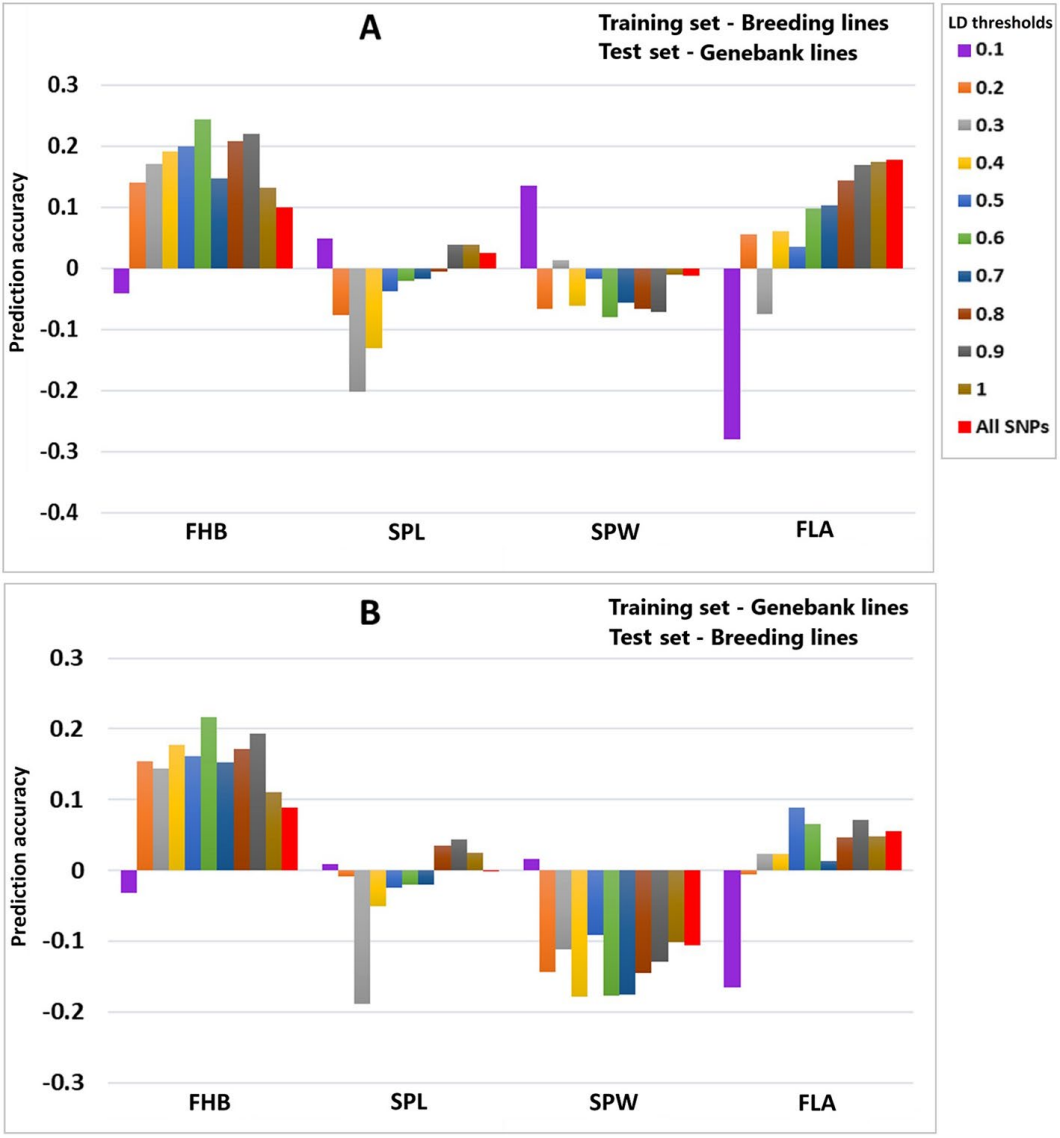
Genomic prediction with haplotype-tagged SNPs across the two independent populations tested in RR-BLUP model. **A** Prediction accuracy of models trained with breeding lines and tested in genebank lines (scenario 6 and 14). **B** Prediction accuracy with the genebank lines used as training and breeding lines as test sets (scenario 7 and 15). *FHB* Fusarium head blight; *SPL* Spike length; *SPW* Spike width; *FLA* Flag leaf area; *LD* Linkage disequilibrium

A principal component analysis of the breeding and genebank lines displayed a distinct population structure (Supplementary Fig. 1) that lead to a low genomic prediction accuracy across the two populations (Scenarios 14 and 15) (Supplementary Table S3). Pre-selection of SNPs via haplotype-tagging (Scenarios 6 and 7) did not help to improve the prediction accuracy of SPL, SPW and FLA. However, tagged SNPs appeared to improve the accuracy for FHB in both training-test directions (Fig. 3). For instance, when the model trained in breeding lines and validated with genebank lines, the prediction accuracy improved from 0.10 with all 6421 non-preselected SNP markers to 0.24 with only 1195 LD pruned (*r*^2^ = 0.6) tagged SNPs (Fig. 3A). Similarly, the accuracy increased from 0.09 to 0.22 through tagged markers pruned at a similar LD threshold with the model trained in genebank lines and validated in the breeding lines (Fig. 3B).

### Trait-linked markers from combined populations

Except in FLA, the first 100 significant SNP markers identified from the combined training-test sets comprising the breeding and genebank lines (Scenario 2) improved the genomic prediction accuracy with 0.45, 0.42 and 0.13 for FHB, SPW and SPL, respectively (Fig. S2). The prediction accuracy dropped when the next 100 SNP markers, viz. significant SNPs located from 101 to 201, fitted in the prediction models of the three traits. The first 200 significant SNPs only improved the prediction accuracy of SPW compared to only using the first 100 SNP markers (Fig. S2). None of the significant SNP sets applied in the models improved the genomic prediction accuracy of the FLA trait.

### Trait-linked SNPs identified only in training population

**Fig. 4:**
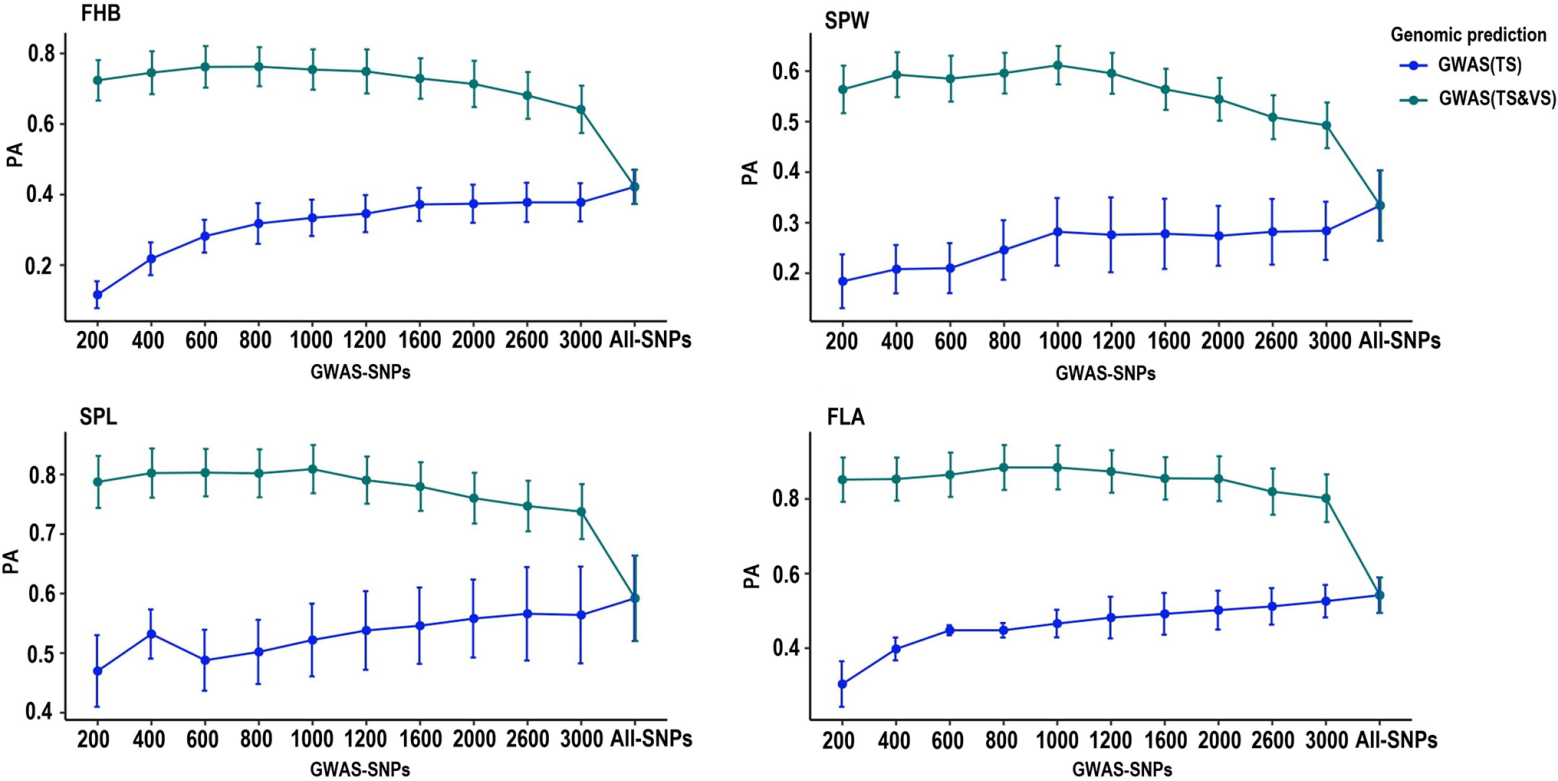
Genomic prediction accuracy with different sets of GWAS-identified trait-linked SNP markers in breeding lines tested in the RR-BLUP model. The genomic prediction accuracy with GWAS-SNPs identified from the combined training-test populations (scenario 5) was compared with those identified only from training populations (scenario 4) to show the inflation when GWAS and GP analysis are non-independent. *FHB* Fusarium head blight; *SPL* Spike length; *SPW* Spike width; *FLA* Flag leaf area; *TS* Training set; *VS* test set; *GP* Genomic prediction; *GWAS* Genome-wide association analysis; *PA* Prediction accuracy

Genomic prediction with trait-linked SNPs identified from the combined training-test sets was compared with predictions only from training set; hence, the former could inflate the accuracy due to the non-independence of the genomic prediction and GWAS analysis methods. Therefore, different sets of trait-linked SNPs were identified only from the training population adapting the five-fold cross-validation scheme using the 272 breeding lines (Scenario 4). The GWAS analysis results including the Manhattan and Q-Q plots conducted following the five-fold cross-validation scheme can be found in Supplementary File 2. The average values from the five genomic prediction analysis iterations revealed that employing the various sets of GWAS-identified trait-linked SNPs did not improve the prediction accuracy of the four tested traits. However, an inflated prediction accuracy was recorded with GWAS-SNPs identified from the combined training-test populations (Scenario 5) in the four traits (Fig. 4). The genomic prediction accuracy improved as the number of trait-linked SNPs increased from 200 to 3000, but the highest was recorded with all SNP markers (Supplementary Table S4).

### Across populations genomic prediction with trait-linked SNPs

**Fig. 5:**
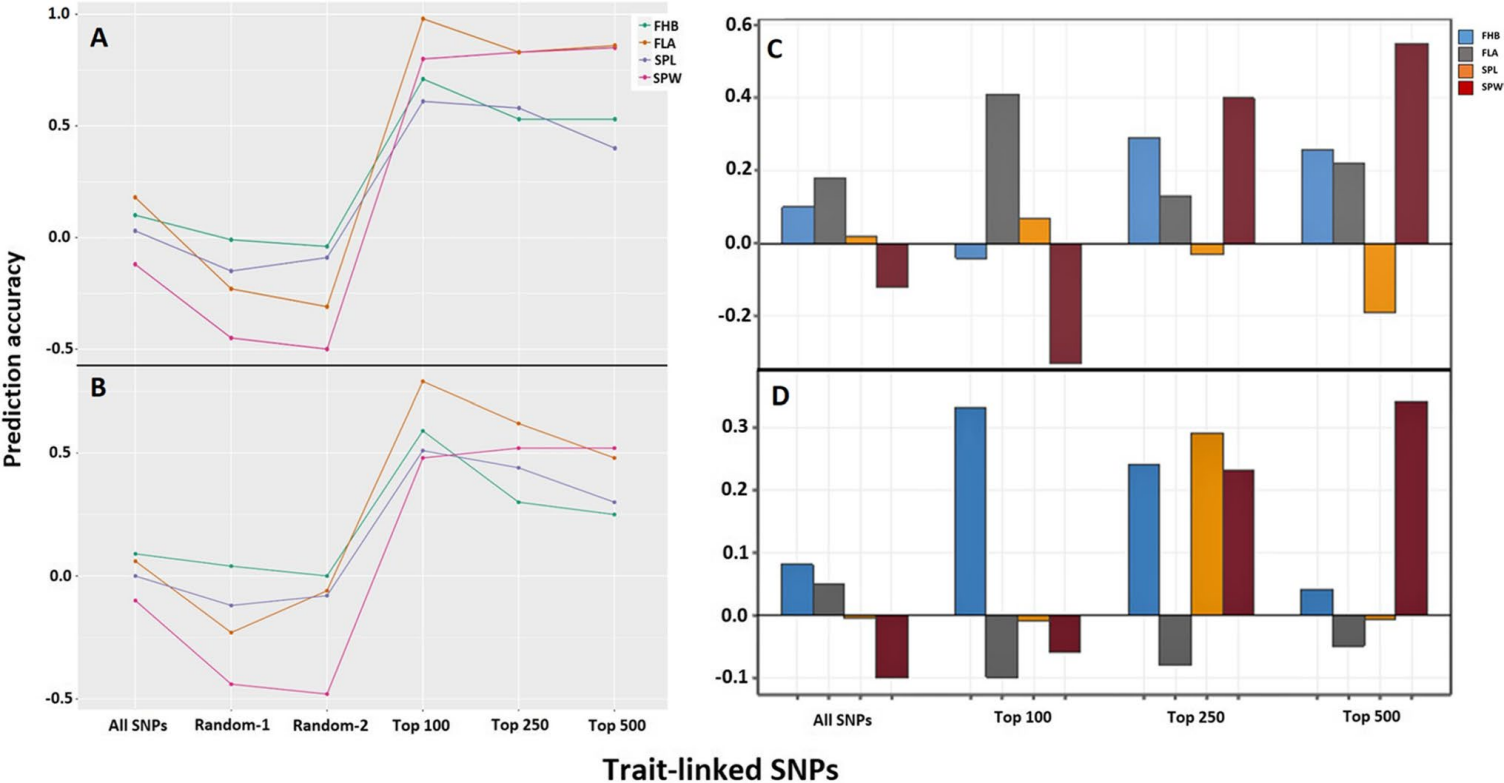
Genomic prediction with trait-linked SNPs across the two independent populations with the RR-BLUP model. In the first case, trait-linked SNPs was identified through GWAS from the two populations combined followed by genomic prediction analysis trained with breeding lines and tested in genebank lines (**A**) (scenario 8); trained with genebank lines and tested in breeding lines (**B**) (scenario 9). In the second case, the GWAS analysis was conducted only from the training population and the genomic prediction model was trained with identified trait-linked SNP markers using the breeding lines and tested in genebank lines (**C**) (scenario 10) and with the other way around (**D**) (scenario 11)

The genomic prediction accuracy across the two independent populations with non-preselected SNPs was very low (Fig. 5). Trait-linked SNPs identified from the combined breeding- genebank lines (Scenarios 8 and 9) improved the prediction accuracy of the four traits significantly regardless of the type of populations used as training and testing populations viz. trained with breeding lines and validated in genebank lines (Fig. 5A) or the other way around (Fig. 5B). However, when trait-linked SNPs were identified only from the training population (Scenarios 10 and 11), preselected markers did not show a similar trend of genomic prediction accuracy improvement across tested traits with different sets of trait-linked markers. For instance, the first 100 significant SNP markers did not improve the genomic prediction accuracy of FHB when the model was trained with breeding lines and validated in genebank lines (Fig. 5C), but a beneficial improvement was recorded when the two populations were used the other way around, improving the accuracy from 0.08 to 0.33 (Fig. 5D). Both the first 250 and 500 SPW-linked SNPs significantly improved the genomic prediction accuracy in both training-test directions with the breeding and genebank lines (Fig. 5C, D).

## Discussion

### Comparing genomic prediction accuracy across models

A moderate to high genomic prediction accuracy was recorded from the current study across six different statistical models, four traits and various sets of preselected and non-preselected SNP markers tested within the combined populations and across independent population cross-validation analysis. From the within population analysis, the five different Bayesian models predicted the GEBVs of FHB and SPL in similar range with the RR-BLUP model but a slightly higher accuracy for SPW. However, RR-BLUP appeared to outperform the Bayesian models for FLA improving the accuracy by 0.33 when non-preselected markers fitted in the model and by 0.29 compared to the highest predicted value scored in the Bayesian ridge regression model using 3090 haplotype-tagged (*r*^2^ = 0.9) SNP markers. Several empirical studies conducted in different crops have shown a slight or non-significant differences in genomic prediction accuracy between statistical models grouped in the Bayesian and BLUB-based approaches (Meher et al. 2022; Merrick and Carter 2021; Roorkiwal et al. 2016; Semagn et al. 2022a; Thavamanikumar et al. 2015; Tsai et al. 2020; Wang et al. 2015). None withstanding, these models could perform differently depending on various factors, such as genetic architecture of the trait (i.e., whether controlled by few or several QTL), sample size, heritability, extent of LD between the marker and QTL, and density of the marker data (Habier et al. 2007; Lorenz et al. 2011; Wang et al. 2015; Zhong et al. 2009).

### Genomic prediction for Fusarium head blight resistance

Fusarium head blight (FHB or scab) is one of the most important fungal diseases of wheat that leads to a substantial loss in grain yield and quality by infecting the spike (Mcmullen et al. 2012). Genotypes resistance to FHB is quantitative in nature, and multiple QTL have been identified with major to minor effects (Liu et al. 2009). Venske et al. (2019) compiled 556 QTL distributed across the three genomes and 21 chromosomes from 76 different studies. The enormous number of identified QTL coupled with other non-identified minor-effect QTL across the entire genome makes marker-assisted selection intricate. Instead, evaluation of individual genotypes with their overall genetic merit toward resistance to the disease through genomic prediction models is a more efficient and feasible approach. In addition, the difficulty of phenotypic screening of the disease in both controlled conditions and field trials (Zhang et al. 2022) makes genomic selection the best option in FHB resistance breeding. Previous studies have shown the potential of genomic prediction in FHB resistance in wheat (Arruda et al. 2015; Dong et al. 2018; Herter et al. 2019; Rutkoski et al. 2012; Verges et al. 2020; Zhang et al. 2022). However, these investigations were a non-haplotype-based genomic prediction analysis and focused on optimizing the accuracy with different sets of training and test combinations via testing in various models and with the inclusion of GxE and other covariates. For instance, Zhang et al. (2022) investigated genomic prediction for percentage of FHB damaged kernels and FHB disease index using 476 elite and advanced winter wheat breeding lines from South Dakota State University, USA, and reported a prediction accuracy between 0.32–0.40 and 0.32–0.42, respectively, from five different models and two years field trials. Rutkoski et al. (2012) reported a genomic prediction accuracy ranging from 0.34 to 0.45 from different models for FHB damaged kernels in 322 winter wheat breeding lines collected from different public and private breeding line across the eastern USA and Canada. With non-preselected SNPs, we predicted slightly higher than these reports for FHB with a range of 0.44–0.46 from the six different models regardless of the several factors that could lead to prediction accuracy differences across these studies such as environment, applied experimental designs, the size and genetic relatedness of the training-test populations. The BRR model with LD-based haplotype-tagged SNP markers improved the accuracy to 0.50, while the accuracy stretched up to 0.90 when the first 100 significantly associated SNPs identified from the combined populations and fitted in the RR-BLUP model. Nonetheless, GWAS was conducted from the combined training-test populations that could lead to an inflated accuracy since the genomic prediction and GWAS analysis were not independent (McGowan et al. 2021). Beneficial genomic prediction accuracy improvements could not be recorded for FHB when the model was trained with various sets of trait-linked SNPs identified only from 80% of the 272 breeding lines and validated with the remaining 20% masked individuals during the GWAS analysis. Hoffstetter et al. (2016) reported a genomic prediction accuracy of 0.35 to FHB resistance when all 4858 SNPs and 28,311 silicoDArT markers fitted to the RR-BLUP model. However, the accuracy increased up to 0.64 when only 1556 most significant markers (*P* < *0.05*) applied in the prediction analysis.

### Genomic prediction with haplotype-tagged SNP markers

The Bayesian-based models were initially proposed to optimize the accuracy of the BLUP-based models since the former apply different prior assumptions for predictors and assign different weights based on their effect, while the latter assume all markers contributes to the trait (De Los Campos et al. 2013; Endelman 2011; Habier et al. 2011). The Bayesian models should especially be robust enough to overcome issues raised from noise from non-effect markers since they either shrink these variants effect toward zero or exclude from the model. However, the current analysis specified that marker reduction via haplotyping could yet improve these models. This indicates one should not solely rely upon models with the built-in type of marker selection applied in Bayesian models and approaches such as haplotype-tagging could be an invaluable tool for selecting markers to leverage genomic prediction accuracy.

The haplotype-based genomic prediction has been widely applied in animal breeding, and results have shown its potential to improve the genomic prediction accuracy of several traits with economic relevance (Cuyabano et al. 2014, 2015; Li et al. 2021; Won et al. 2020). However, the method is not commonly applied in plant breeding and very few studies have shown its potential to optimize prediction models (Bhat et al. 2021; Matias et al. 2017). Werner et al. (2018) applied pre-selection of SNPs via LD-based haplotype-tagging at *r*^2^ > 0.8 and selected markers reduced into subsets of tagged SNPs from 9793 to 50 for genomic prediction of six different traits in Asian semi-winter rapeseed diversity panel. They concluded that marker reduction via tagging could improve the prediction accuracy and can be used as a cost-effective genotyping tool which is easily producible from available high-density SNP arrays. The current study exploited haplotype-based SNP tagging with ten selected LD levels aimed at finding the optimum threshold to predict GEBVs of four different traits with highest accuracy. Except for SPW, results from the combined populations indicated several different LD thresholds are required to capture the optimum genomic prediction accuracy across traits and statistical models. However, with only breeding lines, most of the optimum genomic prediction accuracy of a particular trait was achieved from a distinct LD threshold across models (Fig. 2). The variation in LD patterns between the breeding lines and genebank genotypes in the combined analysis could have risen due to the fact that two populations have distinct genetic background making a distinct population structure. The advanced breeding lines developed from Lantmännen made the first group while the other comprised predominantly cultivars with some historical landraces preserved in Nordgen represented a century of winter wheat breeding history of the Scandinavian region (Odilbekov et al. 2019). Flint-Garcia et al. (2003) pointed out that the extent of LD is strongly influenced by population structure and the origin and/or genetic basis of genotypes. Previous study has shown a higher level of heterogeneity in the extent of LD across the wheat genome and the LD between neighboring SNPs and size of haplotype blocks were higher in cultivars than landraces (Cavanagh et al. 2013).

In the current study, with the two populations combined, the majority of highest prediction accuracy with haplotype-tagged SNPs was obtained with LD thresholds ranged from *r*^2^ = 0.3 to 0.8, whereas the LD thresholds at the two extreme sides did not lead to an improved genomic prediction accuracy. This is because increasing haplotype length could improve the probability of capturing the LD between markers in blocks with QTL of a particular trait (Sallam et al. 2020). However, a highly stretched length of haplotypes could severely reduce the number of variables in the model, which ultimately lead to a reduced prediction accuracy (Hess et al. 2017). Ben Hassen et al. (2018) reported an overall LD level of 0.49–0.64 resulted in higher prediction accuracy for three agronomic traits in rice advanced inbred accessions. Notwithstanding, with breeding lines, four different models achieved their highest genomic prediction accuracy of SPW with the weakest applied LD threshold (*r*^2^ = 0.1, 566 tagged-SNPs) while the other two reached their peak at *r*^2^ of 0.2 and 0.3 using 899 and 1171 haplotype-tagged SNPs, respectively. A previous simulation study conducted in animals suggested an LD at* r*^2^ = 0.2 as optimum threshold for haplotype-based genomic prediction (Calus et al. 2008). Overall, our study has revealed that the linkage disequilibrium thresholds for optimum genomic prediction accuracy varied across traits. Both SPW and FHB required weak LD (*r*^2^ < 0.5) to achieve the highest genomic prediction accuracy while a stringent LD thresholds was suitable for SPL and FLA across the six tested prediction models.

In this study, the five Bayesian models exhibited a slightly different performance across the various haplotype-tagged SNP markers. For instance, the BRR model performed better than other Bayesian models with haplotype-tagged SNPs and had the highest prediction accuracy for FHB, SPL and FLA. Ballesta et al. (2019) reported an increased accuracy with haplotype-based genomic prediction for low-heritability traits in *Eucalyptus globulus* (Labill.) and pointed out that the BRR model performed better with haplotypes compared to the other Bayesian models. Matias et al. (2017) reported haplotype-based genomic prediction for grain yield and plant height in maize improved the accuracy significantly compared to SNP-based prediction but did not increase in rice for the same traits. In contrary, Jiang et al. (2018) stated that haplotype-based GBLUP model improved the accuracy of protein content and flowering time in rice but failed to give any significant benefits to the five different agronomic traits tested in a maize panel. He et al. (2019) indicated a significant prediction improvement with haplotype-based genomic prediction for wheat screenings percentage and grain yield with haplotype blocks constructed with 3–10 and 2–4 SNP markers, respectively. Sallam et al. (2020) stated a constant prediction accuracy improvement was recorded with multi-allelic haplotype-based genomic prediction models constructed from 15 adjacent SNP markers in yield and quality-related traits of hard red spring wheat tested in multi-environments. However, these studies used haplotype alleles constructed from all SNPs in haplotype blocks unlike the current study that were represented with single haplotype-tagged SNPs.

### Trait-linked SNP markers for genomic prediction

GWAS-assisted genomic prediction to fit the most significant SNP markers as fixed-effect covariates has been a common method to optimize the prediction accuracy of several traits in crop plants (Alemu et al. 2021a; Gaikpa et al. 2021; Juliana et al. 2022; Moore et al. 2017; Odilbekov et al. 2019; Semagn et al. 2022b; Spindel et al. 2016). However, this method accompanied with some setbacks. The overlapping of the GWAS and genomic prediction validation populations that leads to an overfitting and inflated genomic prediction accuracy is the most common shortcoming (Wray et al. 2013). Moreover, a simulation study showed that trait-linked SNP markers fitted as fixed effect could only improve the genomic prediction accuracy when they are linked to major effect QTL accounting ≥ 10% of the total genetic variance and few major genes present to the target trait (Bernardo 2014). Fitting few major effect SNP markers as fixed and treat others as random effect could yet accompanied with problems raised from marker dimensionality-related issues. Excluding the non-effect SNP markers and utilize only markers significantly linked to a trait on the training population is the other option to predict the breeding values. This approach could alleviate several of the aforementioned hindrances arising from merely fitting the most significant SNP markers as a fixed effect. Recent studies have revealed applying the most significant markers in the model improved the genomic prediction accuracy (Filho et al. 2019; Ling et al. 2021; Tan and Ingvarsson 2022). In addition, selection of trait-linked SNP markers through association analysis or other machine learning methods in genomic prediction analysis is a commonly applied method in animal breeding (Li et al. 2018; Veerkamp et al. 2016; Zhang et al. 2014). The current study discovered significant prediction improvements with the genomic prediction model trained with the first 100 significantly linked SNP markers identified from the pooled training-validation populations. Except for FLA, the other traits prediction accuracy improved by 0.45, 0.42 and 0.13 for FHB, SPW and SPl, respectively. However, the GWAS analysis was conducted from the combined breeding and genebank lines comprising both the training and validation sets. This phenomenon usually leads an inflated prediction accuracy since the two analysis methods were non-independent (McGowan et al. 2021). To circumvent this, trait-linked markers were identified from only training population of breeding lines following the five-fold cross-validation scheme masking 20% of the population used to validate the prediction. Then, different sets of trait-linked markers ranged from 200 to 3000 significant SNPs were used to predict the GEBVs of the four traits. However, any of tested sets of trait-linked markers did not improve the genomic prediction accuracy of the four traits.

### Genomic prediction with genetically distant training and test populations

The other aim of the current study was to evaluate the performance of preselected SNPs with distantly related training and test genotypes. For this purpose, the 272 breeding lines and 147 genebank lines procured from different sources were used as independent training and test sets. The 80–20% training-test cross-validation analysis after merging the breeding and genebank lines appeared to lead a moderate to high genomic prediction accuracy for the four tested traits. The maximum prediction accuracy was 0.42, 0.50, 0.59 and 0.80 for SPW, FHB, FLA and SPL, respectively, across the six models and haplotype-tagged SNP markers. However, treating genebank lines as a training population to predict the GEBVs of FHB and the other three yield-related traits in advanced breeding lines lead to a very low genomic prediction accuracy. Efforts to estimate the GEBVs of 147 genebank lines from the genomic prediction model trained with 272 breeding lines did not show any significant improvement, and the highest prediction accuracy was only 0.18 for FLA. The higher genetic dissimilarity coupled with strong population structure probably contributed to a low genomic prediction accuracy when the two populations used as independent training and test sets as supported in previous studies (Lozada et al. 2019; Michel et al. 2021; Norman et al. 2018). Pre-selection of SNP markers through trait-dependent linked SNPs with GWAS and trait-independent haplotype-tagging did not show a promising improvement in prediction accuracy across the two populations, and genetic relatedness remains the key factor for a successful genomic prediction model.

## Conclusion

The current study applied pre-selection of SNPs for genomic prediction through trait-independent haplotyping followed by SNP-tagging and trait-linked SNPs identification via GWAS analysis. The haplotype-tagged SNPs increased the genomic prediction accuracies of tested traits in six different models. The level of LD thresholds required to prune tagged SNPs for optimum prediction accuracy varied across the four different traits. Genomic prediction models exhibited a stable LD threshold for the topmost prediction accuracy of a particular trait in breeding lines compared to the genebank-breeding lines combined analysis. The trait-linked SNP marker-assisted genomic prediction could be efficient only when individuals on the training and test population are genetically very close. Overall, marker pre-selection methodologies would be invaluable methods in genomic selection as the numbers of SNP markers are expected to increase due to the advancement of whole genome sequencing methods. Furthermore, the haplotyping method could pave the way to a potential low-cost genotyping method for breeders via customized platforms targeting a few key SNP markers tagged to important haplotype blocks.

## Supplementary Information

Below is the link to the electronic supplementary material.

Supplementary file1 (XLSX 13 KB)

Supplementary file2 (XLSX 16 KB)

Supplementary file3 (XLSX 13 KB)

Supplementary file4 (XLSX 11 KB)

Supplementary file5 (ZIP 2025 KB)

Supplementary file6 (ZIP 14628 KB)

Supplementary file7 (DOCX 1174 KB)

## Data Availability

The datasets generated for this study are included in the article and as supplementary files.
