# Supplementary figures and images for: Haplotype-tagged SNPs improve genomic prediction accuracy for Fusarium head blight resistance and yield-related traits in wheat
Source: Theor Appl Genet. 2023 Apr 3;136(4):92. doi: 10.1007/s00122-023-04352-8 (PMC10068637; doi:10.1007/s00122-023-04352-8)

# FarmCPU.FHB\_BLUP

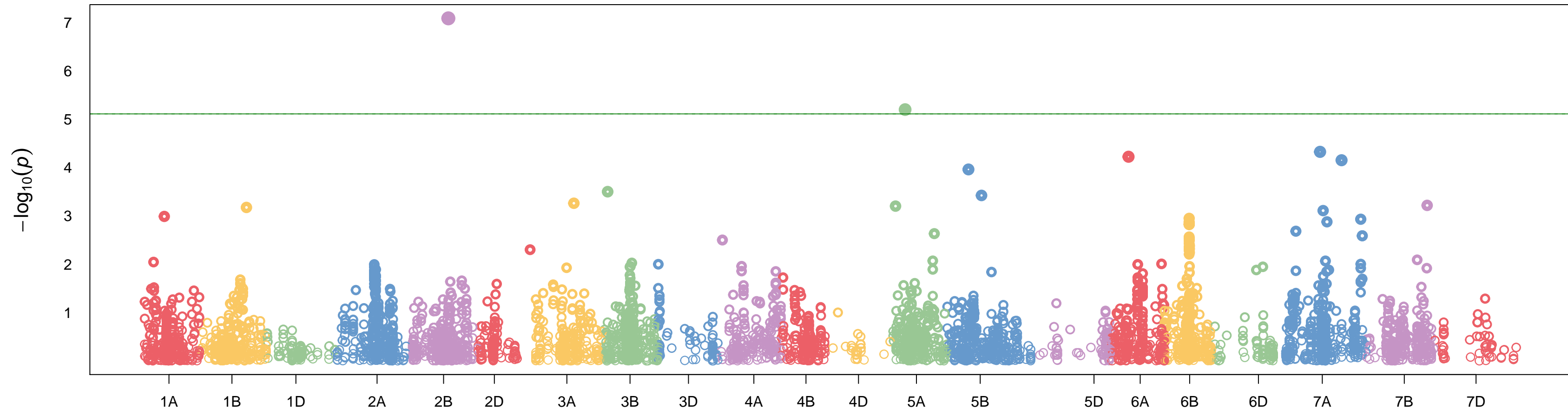

Supplement: Supplementary file 6 — Supplementary file6 (ZIP 14628 KB) [file 122_2023_4352_MOESM6_ESM.zip › FHB/1-ManhattanPlot-FHB.pdf]

# FarmCPU.FHB\_BLUP

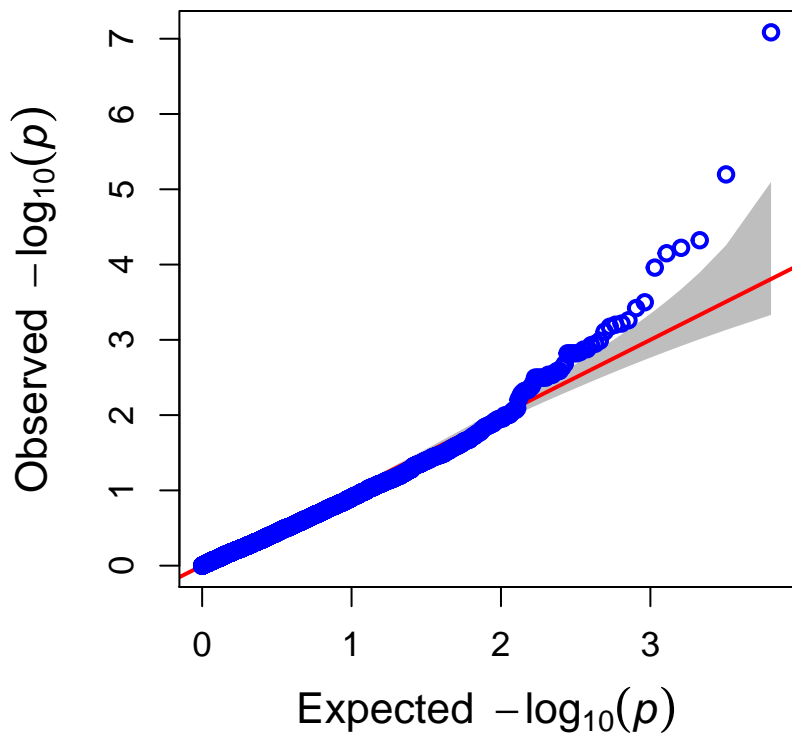

Supplement: Supplementary file 6 — Supplementary file6 (ZIP 14628 KB) [file 122_2023_4352_MOESM6_ESM.zip › FHB/1-QQ-Plot-FHB.pdf]

# FarmCPU.FHB\_BLUP

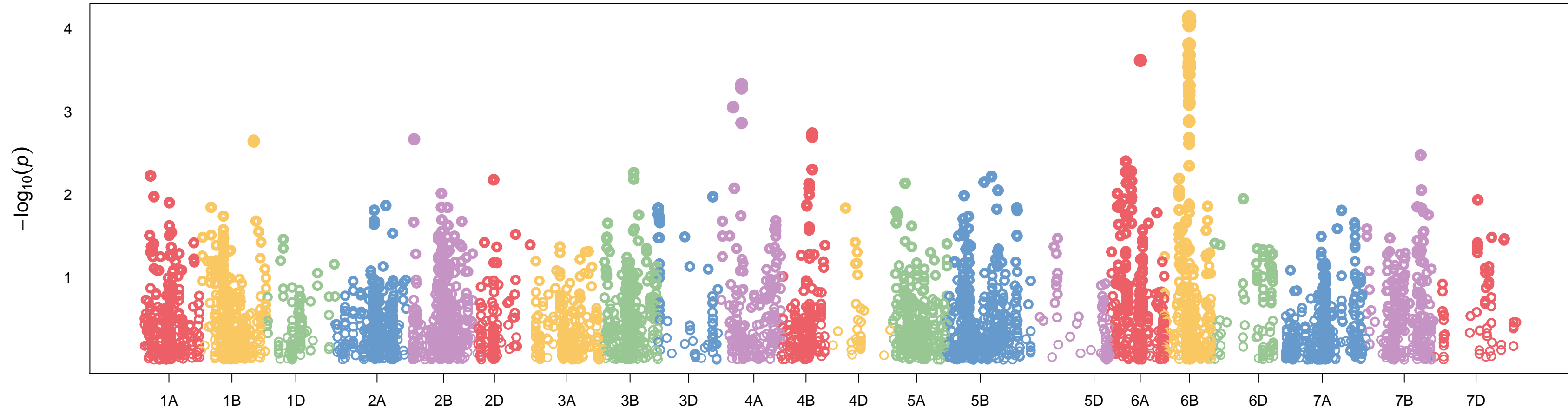

Supplement: Supplementary file 6 — Supplementary file6 (ZIP 14628 KB) [file 122_2023_4352_MOESM6_ESM.zip › FHB/2-ManhattanPlot-FHB.pdf]

# FarmCPU.FHB\_BLUP

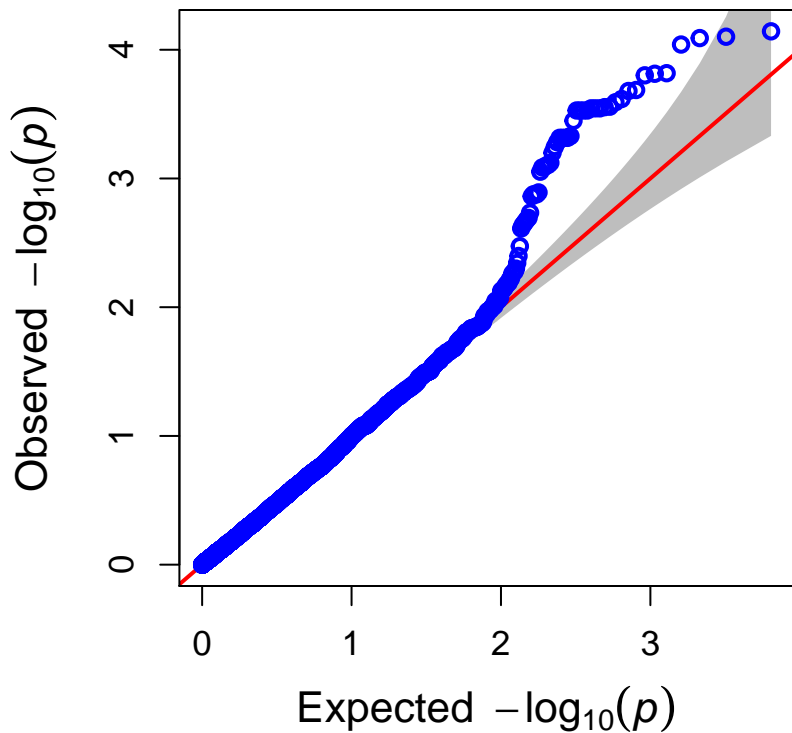

Supplement: Supplementary file 6 — Supplementary file6 (ZIP 14628 KB) [file 122_2023_4352_MOESM6_ESM.zip › FHB/2-QQ-Plot-FHB.pdf]

# FarmCPU.FHB\_BLUP

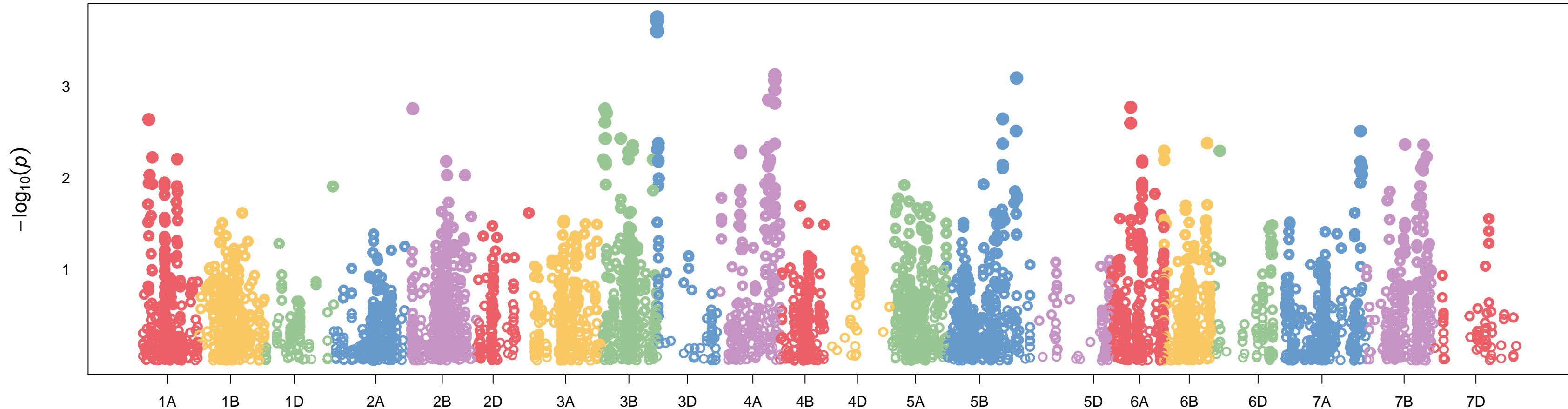

Supplement: Supplementary file 6 — Supplementary file6 (ZIP 14628 KB) [file 122_2023_4352_MOESM6_ESM.zip › FHB/3-ManhattanPlot-FHB.pdf]

# FarmCPU.FHB\_BLUP

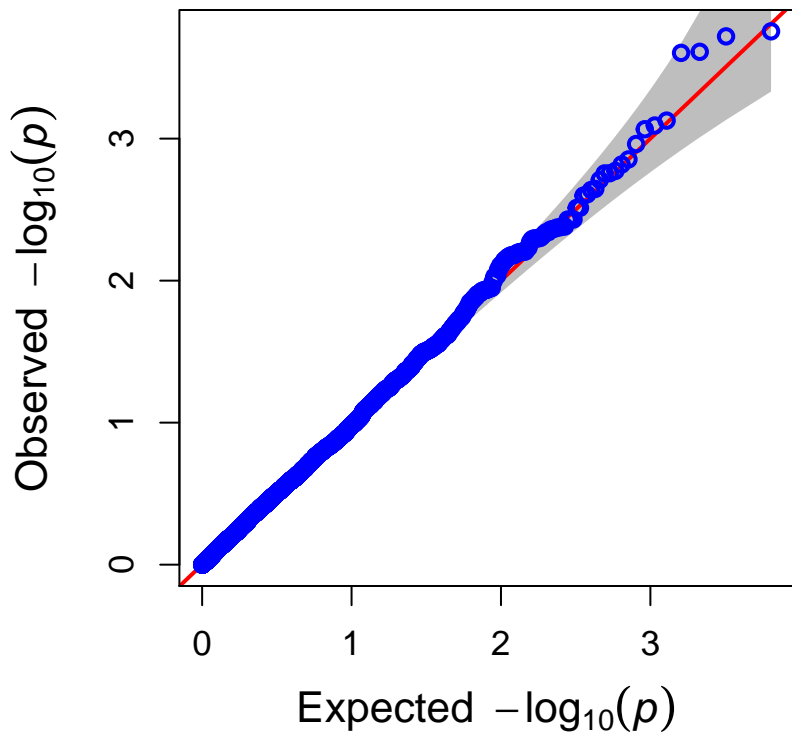

Supplement: Supplementary file 6 — Supplementary file6 (ZIP 14628 KB) [file 122_2023_4352_MOESM6_ESM.zip › FHB/3-QQ-Plot-FHB.pdf]

# FarmCPU.FHB\_BLUP

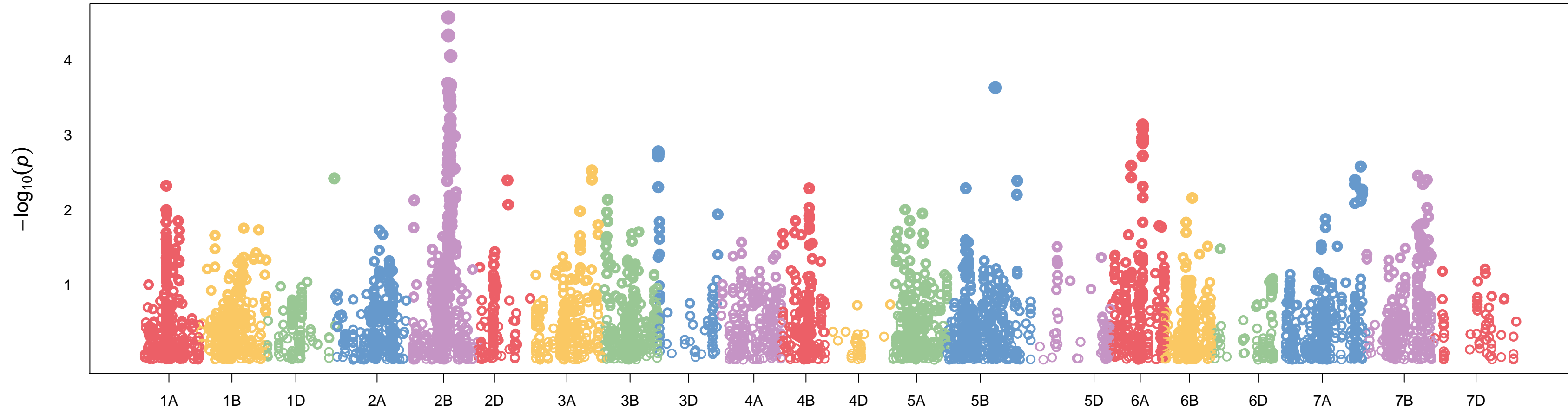

Supplement: Supplementary file 6 — Supplementary file6 (ZIP 14628 KB) [file 122_2023_4352_MOESM6_ESM.zip › FHB/4-ManhattanPlot-FHB.pdf]

# FarmCPU.FHB\_BLUP

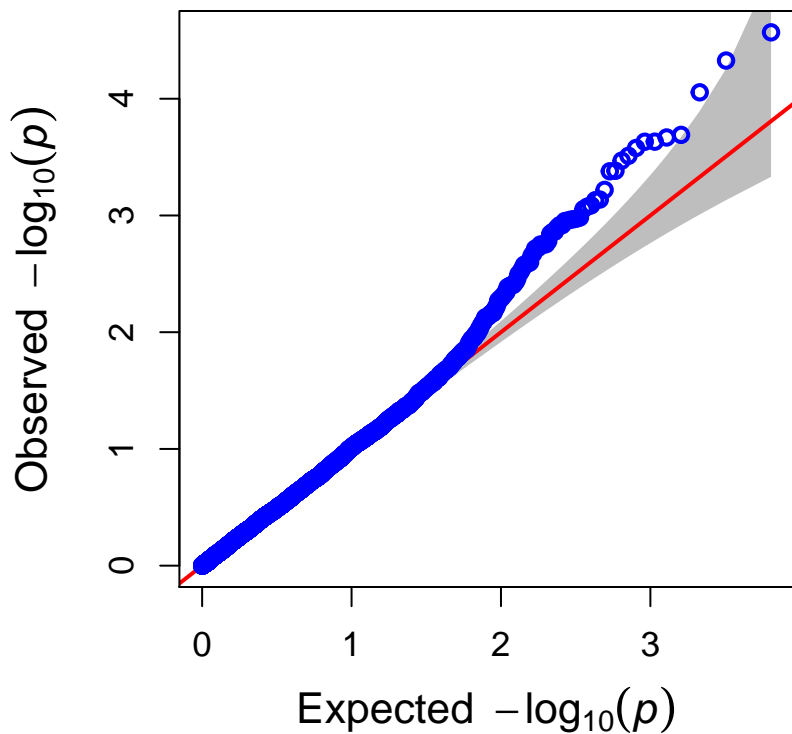

Supplement: Supplementary file 6 — Supplementary file6 (ZIP 14628 KB) [file 122_2023_4352_MOESM6_ESM.zip › FHB/4-QQ-Plot-FHB.pdf]

# FarmCPU.FHB\_BLUP

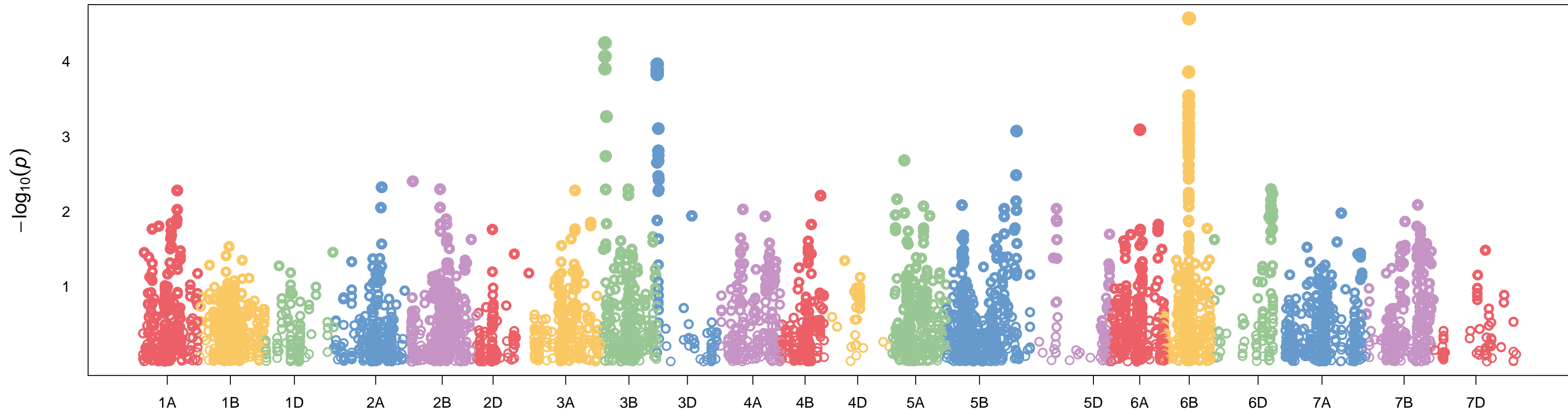

Supplement: Supplementary file 6 — Supplementary file6 (ZIP 14628 KB) [file 122_2023_4352_MOESM6_ESM.zip › FHB/5-ManhattanPlot-FHB.pdf]

# FarmCPU.FHB\_BLUP

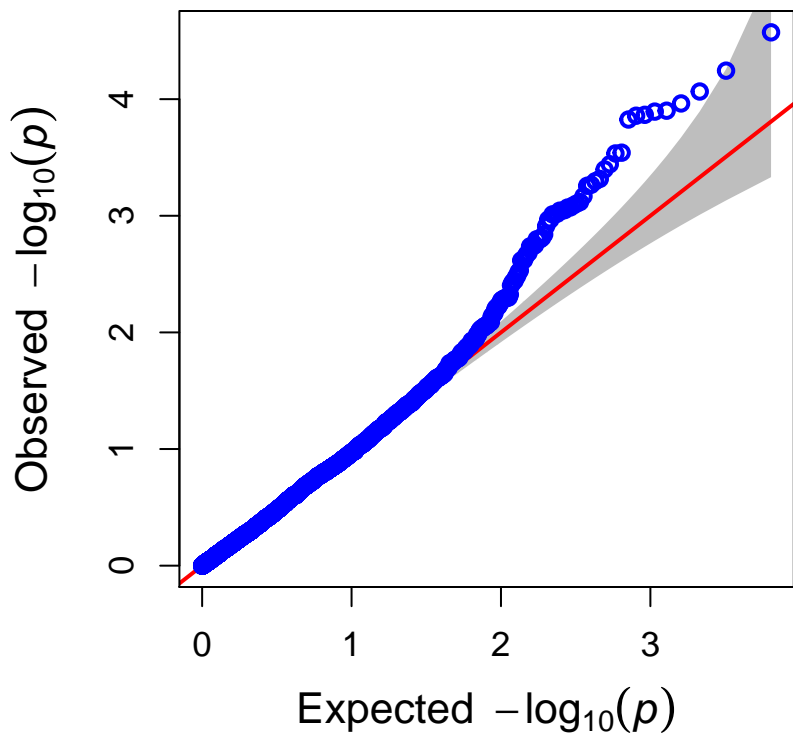

Supplement: Supplementary file 6 — Supplementary file6 (ZIP 14628 KB) [file 122_2023_4352_MOESM6_ESM.zip › FHB/5-QQ-Plot-FHB.pdf]

# FarmCPU.BLUP\_FL A

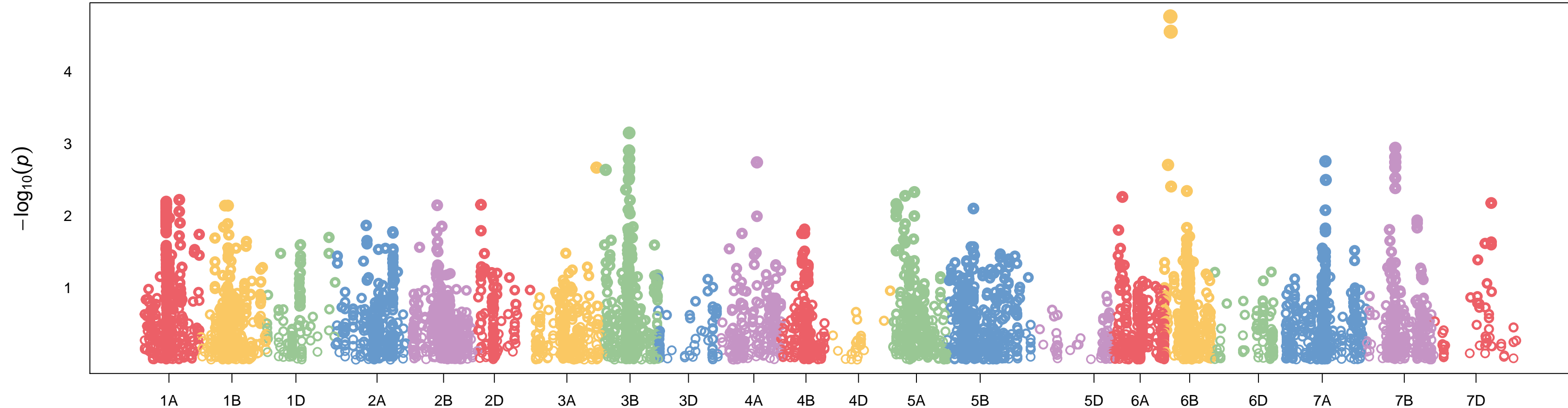

Supplement: Supplementary file 6 — Supplementary file6 (ZIP 14628 KB) [file 122_2023_4352_MOESM6_ESM.zip › FLA/1-Plot.Genomewise-FLA.pdf]

# FarmCPU.BLUP\_FLA

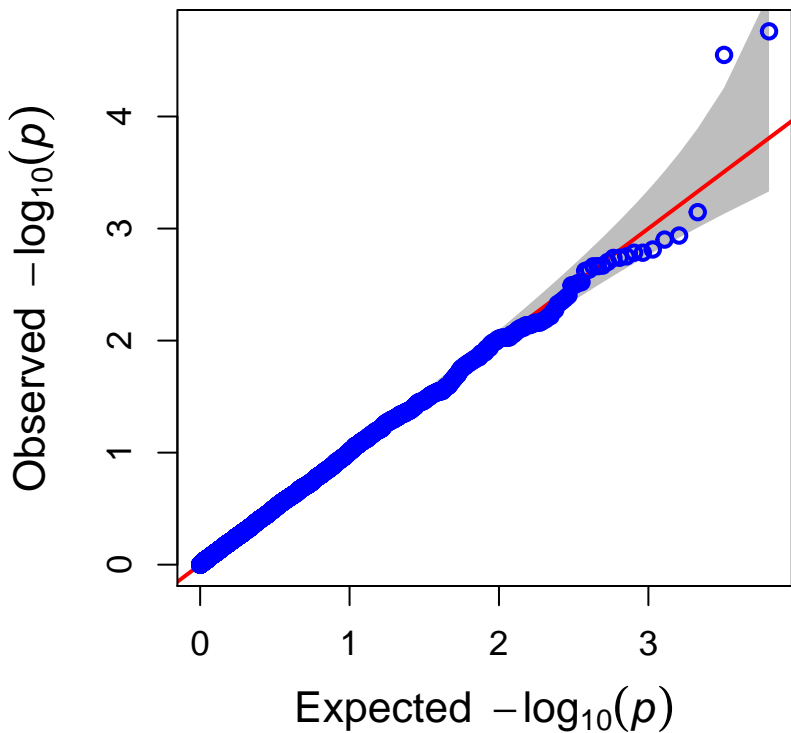

Supplement: Supplementary file 6 — Supplementary file6 (ZIP 14628 KB) [file 122_2023_4352_MOESM6_ESM.zip › FLA/1-QQ-Plot-FLA.pdf]

# FarmCPU.BLUP\_FL A

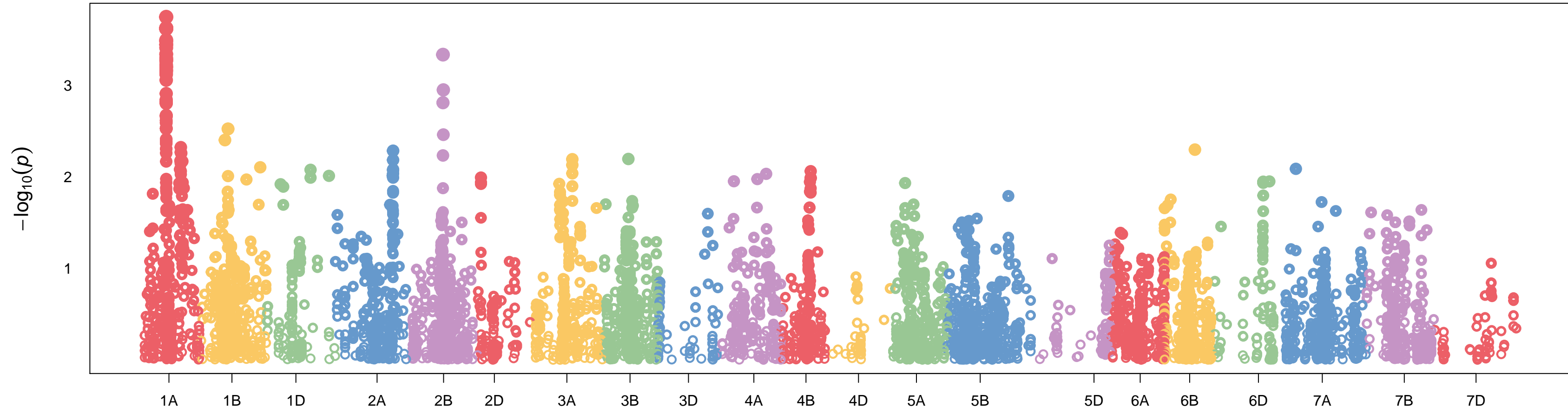

Supplement: Supplementary file 6 — Supplementary file6 (ZIP 14628 KB) [file 122_2023_4352_MOESM6_ESM.zip › FLA/2-Plot.Genomewise-FLA.pdf]

# FarmCPU.BLUP\_FL A

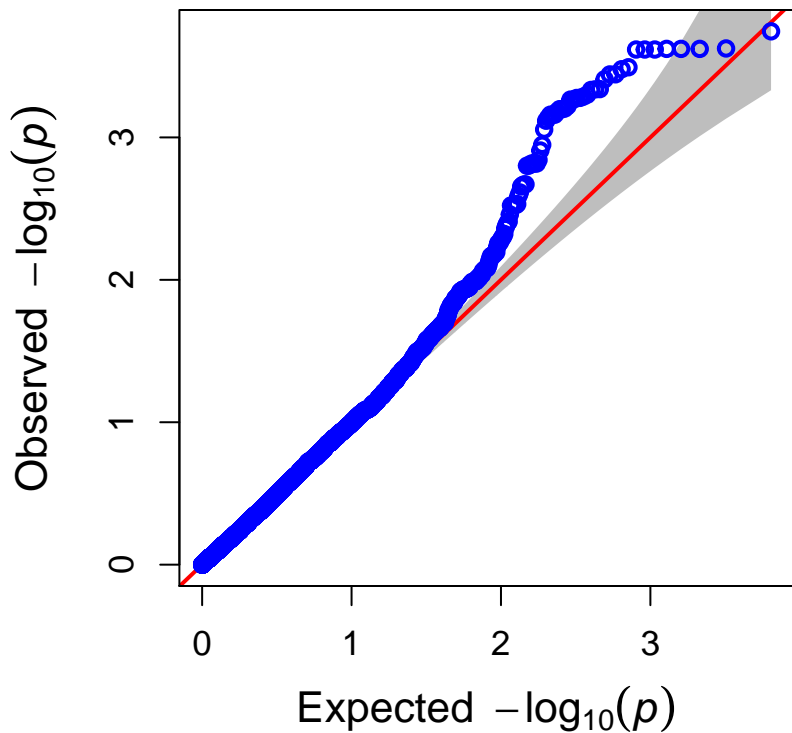

Supplement: Supplementary file 6 — Supplementary file6 (ZIP 14628 KB) [file 122_2023_4352_MOESM6_ESM.zip › FLA/2-QQ-Plot-FLA.pdf]

# FarmCPU.BLUP\_FLA

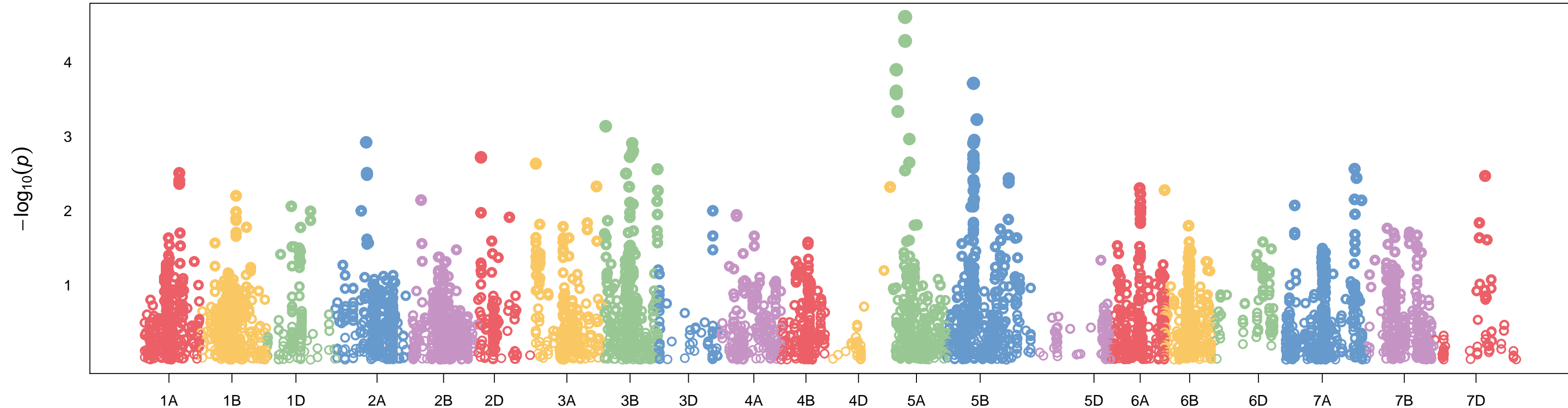

Supplement: Supplementary file 6 — Supplementary file6 (ZIP 14628 KB) [file 122_2023_4352_MOESM6_ESM.zip › FLA/3-Plot.Genomewise-FLA.pdf]

# FarmCPU.BLUP\_FLA

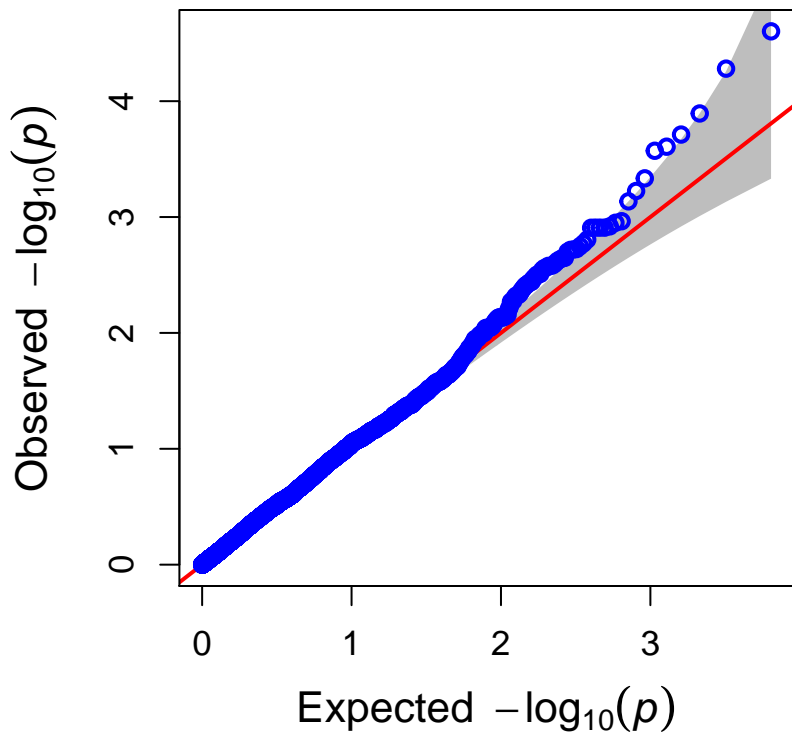

Supplement: Supplementary file 6 — Supplementary file6 (ZIP 14628 KB) [file 122_2023_4352_MOESM6_ESM.zip › FLA/3-QQ-Plot-FLA.pdf]

# FarmCPU.BLUP\_FLA

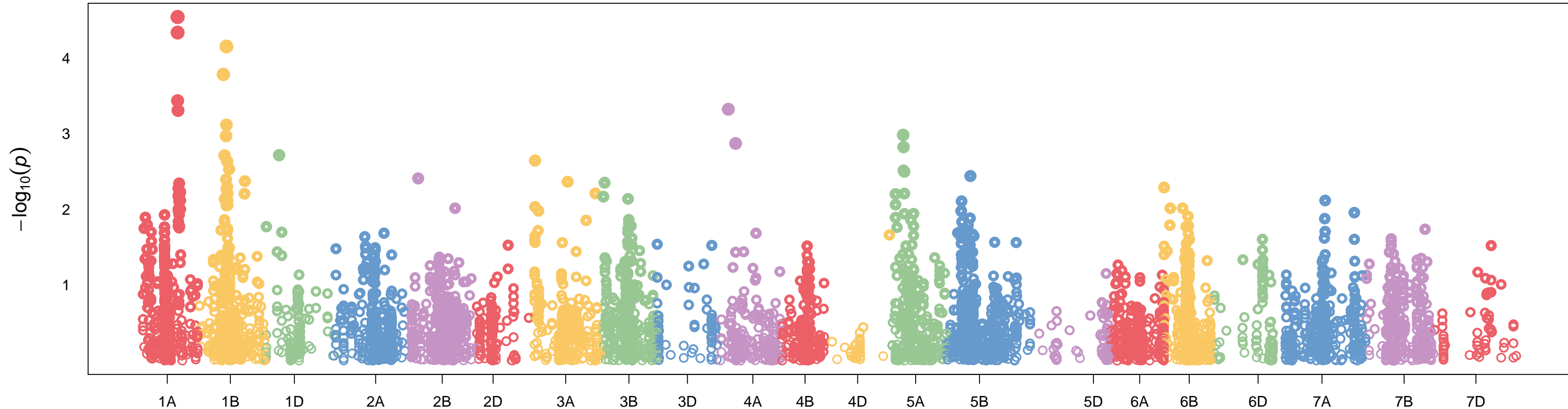

Supplement: Supplementary file 6 — Supplementary file6 (ZIP 14628 KB) [file 122_2023_4352_MOESM6_ESM.zip › FLA/4-Plot.Genomewise-FLA.pdf]

# FarmCPU.BLUP\_FL A

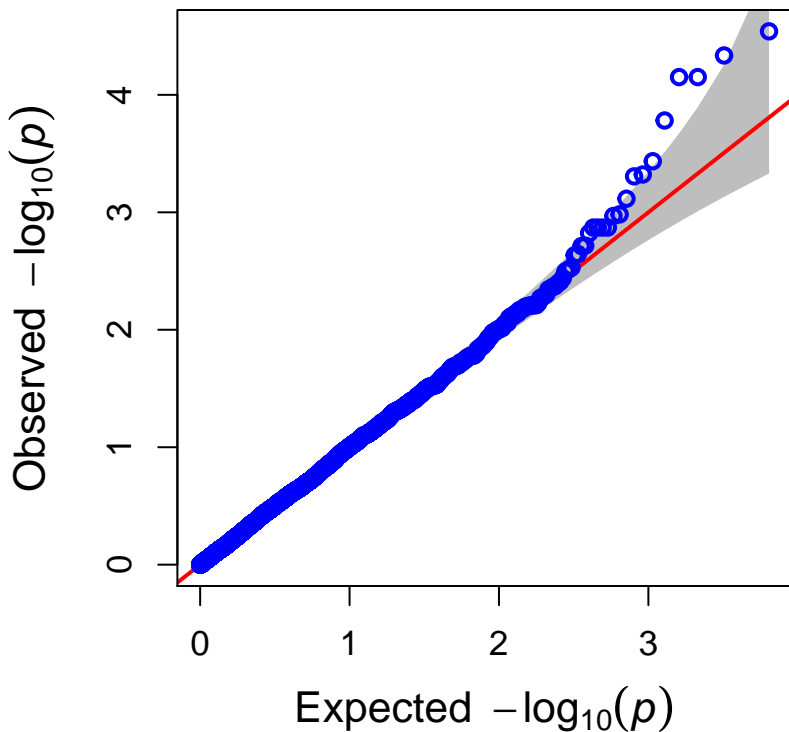

Supplement: Supplementary file 6 — Supplementary file6 (ZIP 14628 KB) [file 122_2023_4352_MOESM6_ESM.zip › FLA/4-QQ-Plot-FLA.pdf]

# FarmCPU.BLUP\_FL A

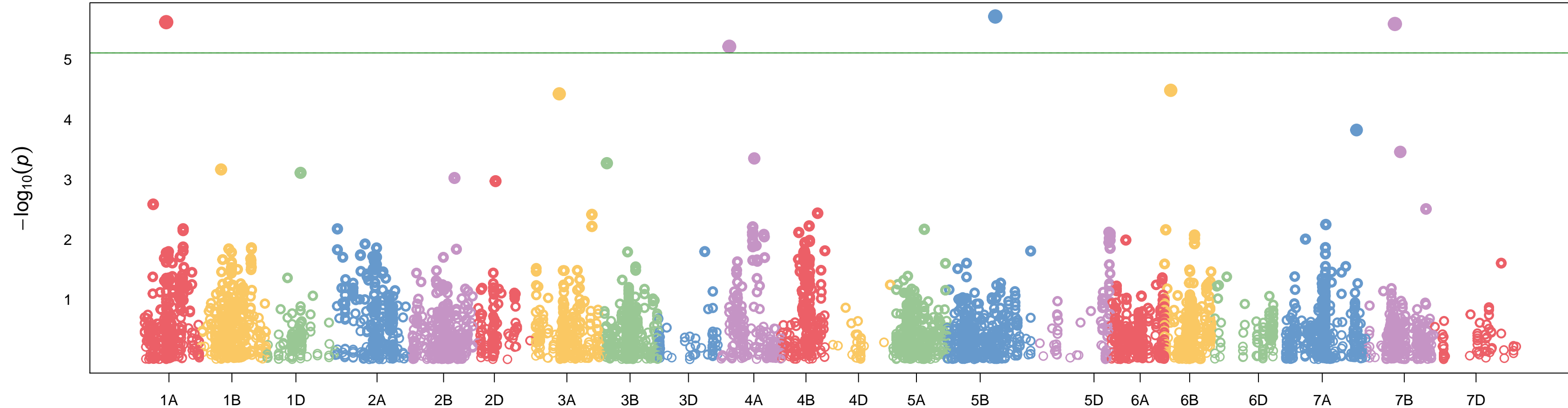

Supplement: Supplementary file 6 — Supplementary file6 (ZIP 14628 KB) [file 122_2023_4352_MOESM6_ESM.zip › FLA/5-Plot.Genomewise-FLA.pdf]

# FarmCPU.BLUP\_FL A

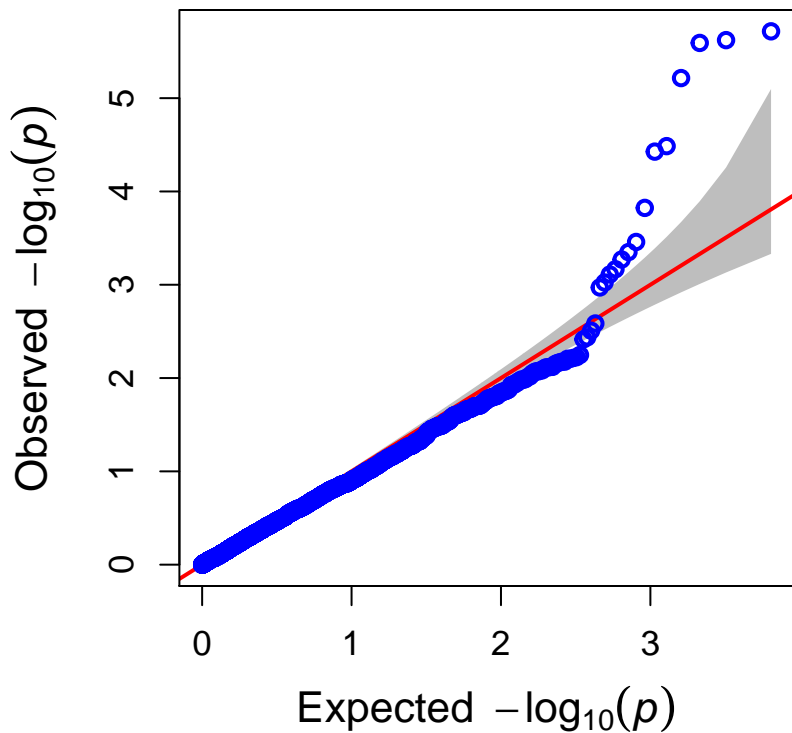

Supplement: Supplementary file 6 — Supplementary file6 (ZIP 14628 KB) [file 122_2023_4352_MOESM6_ESM.zip › FLA/5-QQ-Plot-SPW.pdf]

# FarmCPU.BLUP\_SPL

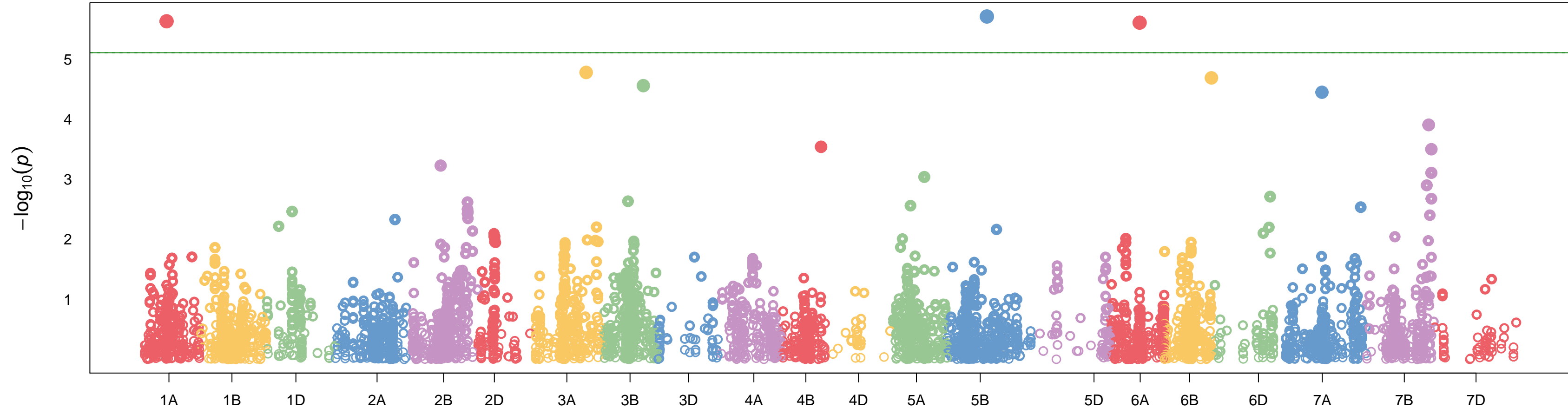

Supplement: Supplementary file 6 — Supplementary file6 (ZIP 14628 KB) [file 122_2023_4352_MOESM6_ESM.zip › SPL/1-Manhattan.Plot-SPL.pdf]

# FarmCPU.BLUP\_SPL

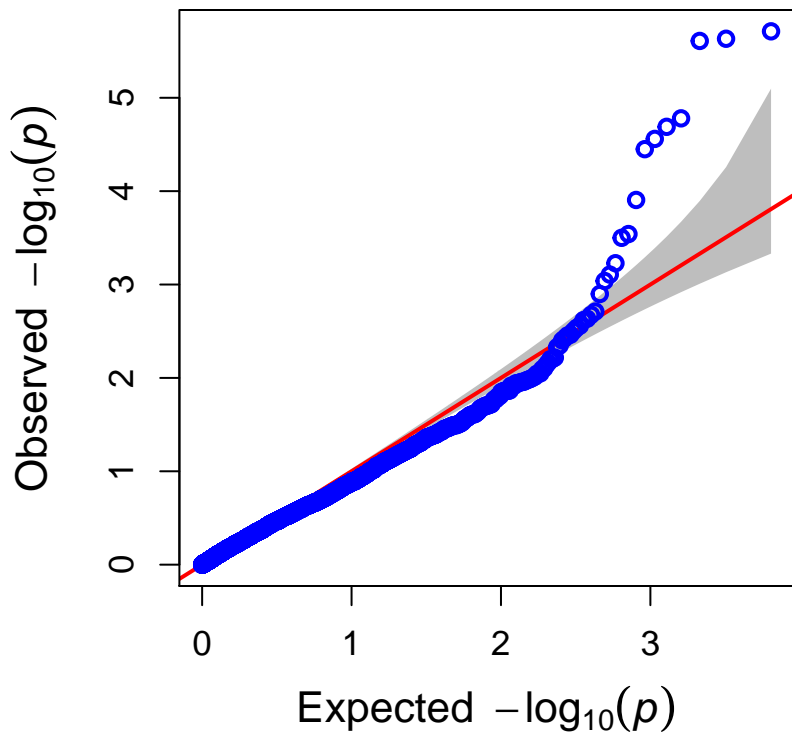

Supplement: Supplementary file 6 — Supplementary file6 (ZIP 14628 KB) [file 122_2023_4352_MOESM6_ESM.zip › SPL/1-QQ-Plot-SPL.pdf]

# FarmCPU.BLUP\_SPL

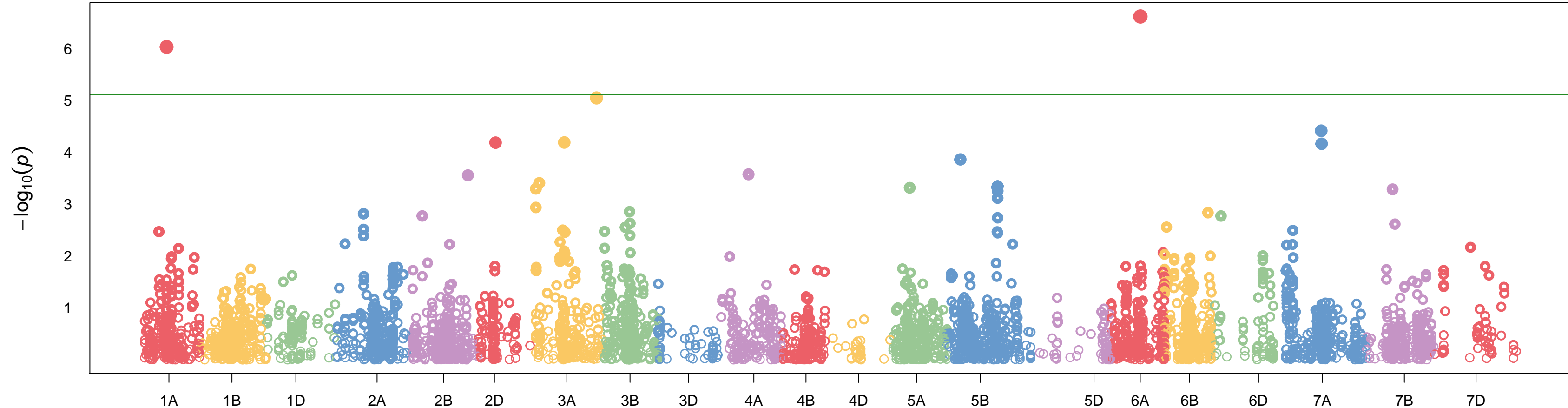

Supplement: Supplementary file 6 — Supplementary file6 (ZIP 14628 KB) [file 122_2023_4352_MOESM6_ESM.zip › SPL/2-Manhattan.Plot-SPL.pdf]

# FarmCPU.BLUP\_SPL

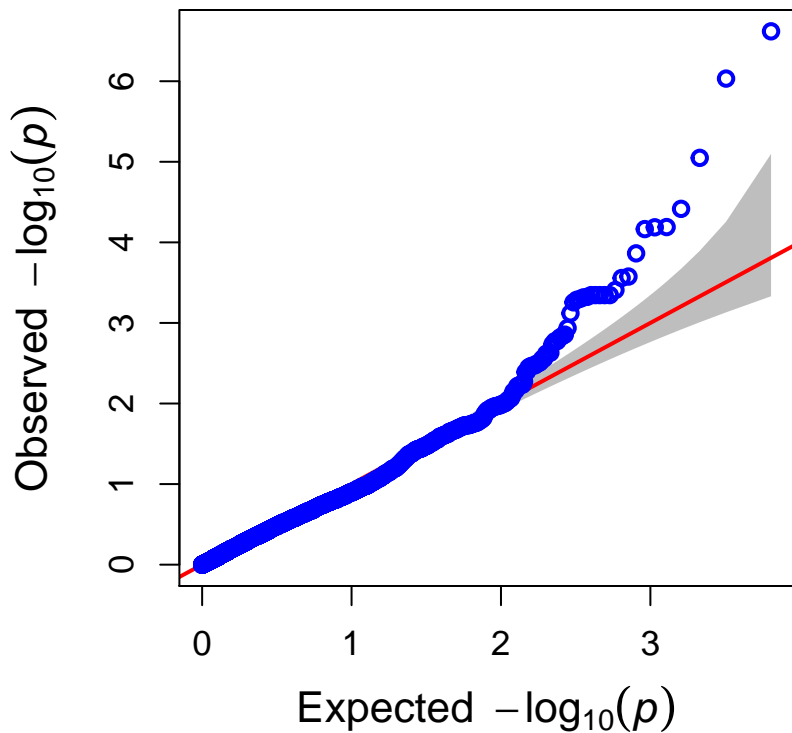

Supplement: Supplementary file 6 — Supplementary file6 (ZIP 14628 KB) [file 122_2023_4352_MOESM6_ESM.zip › SPL/2-QQ-Plot-SPL.pdf]

# FarmCPU.BLUP\_SPL

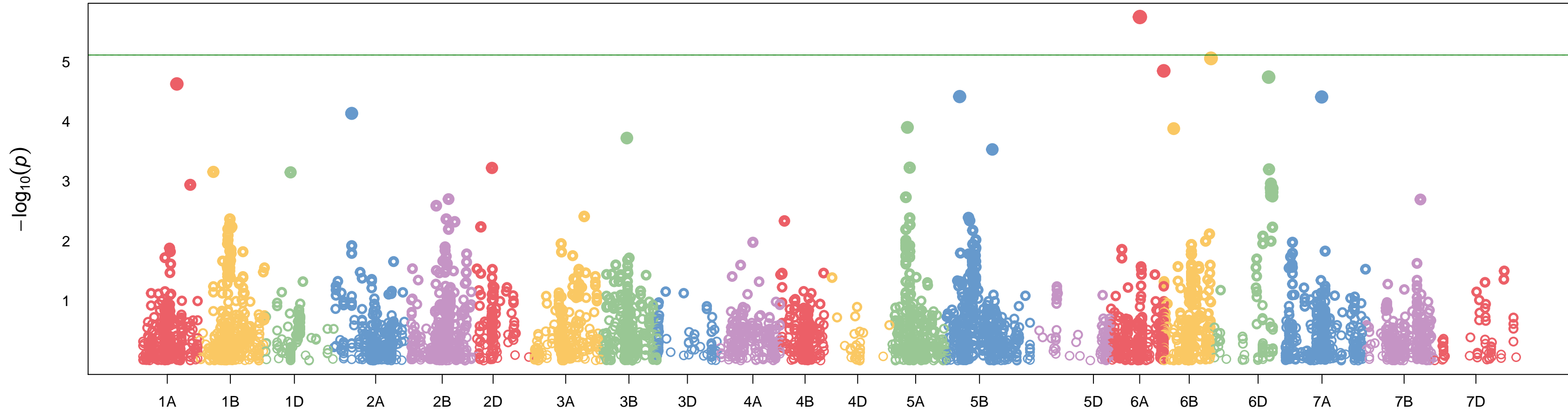

Supplement: Supplementary file 6 — Supplementary file6 (ZIP 14628 KB) [file 122_2023_4352_MOESM6_ESM.zip › SPL/3-Manhattan.Plot-SPL.pdf]

# FarmCPU.BLUP\_SPL

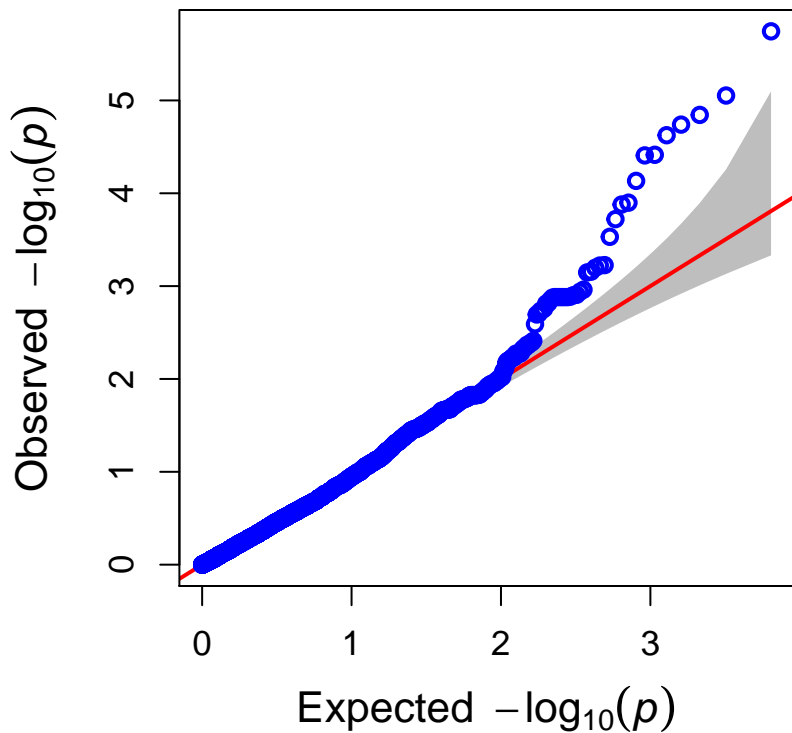

Supplement: Supplementary file 6 — Supplementary file6 (ZIP 14628 KB) [file 122_2023_4352_MOESM6_ESM.zip › SPL/3-QQ-Plot-SPL.pdf]

# FarmCPU.BLUP\_SPL

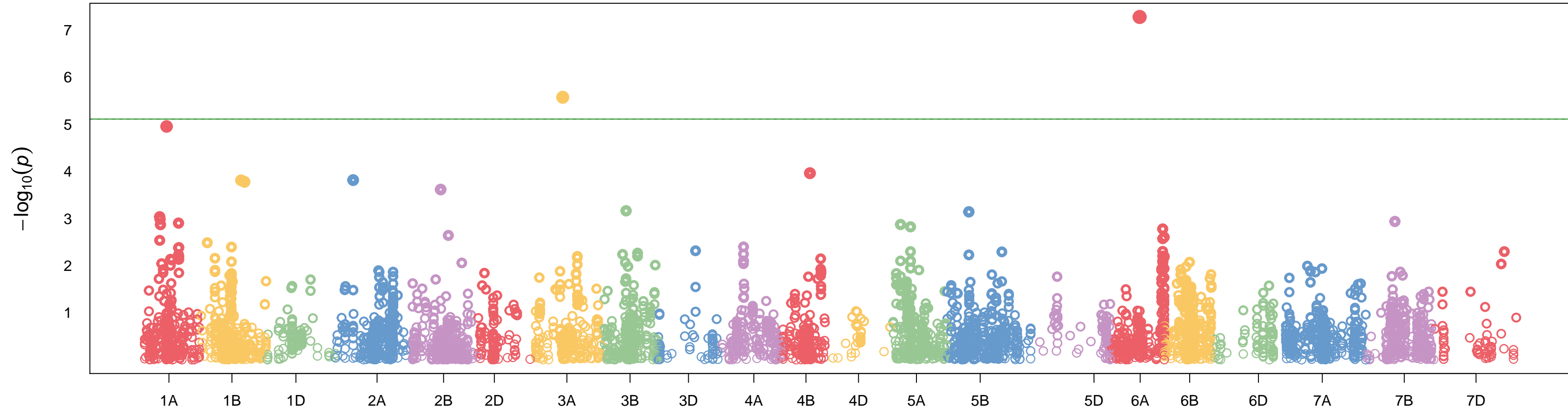

Supplement: Supplementary file 6 — Supplementary file6 (ZIP 14628 KB) [file 122_2023_4352_MOESM6_ESM.zip › SPL/4-Manhattan.Plot-SPL.pdf]

# FarmCPU.BLUP\_SPL

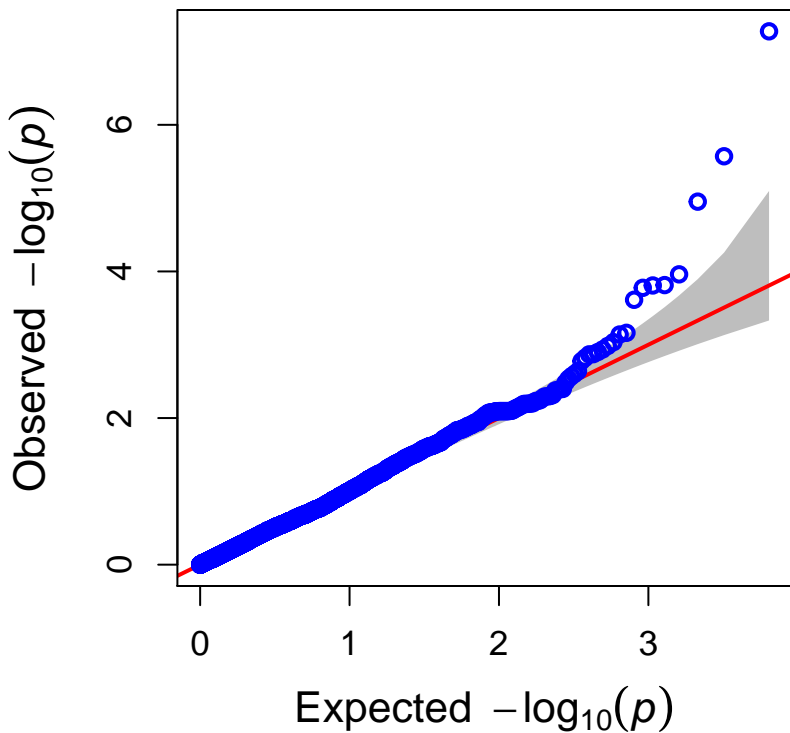

Supplement: Supplementary file 6 — Supplementary file6 (ZIP 14628 KB) [file 122_2023_4352_MOESM6_ESM.zip › SPL/4-QQ-Plot-SPL.pdf]

# FarmCPU.BLUP\_SPL

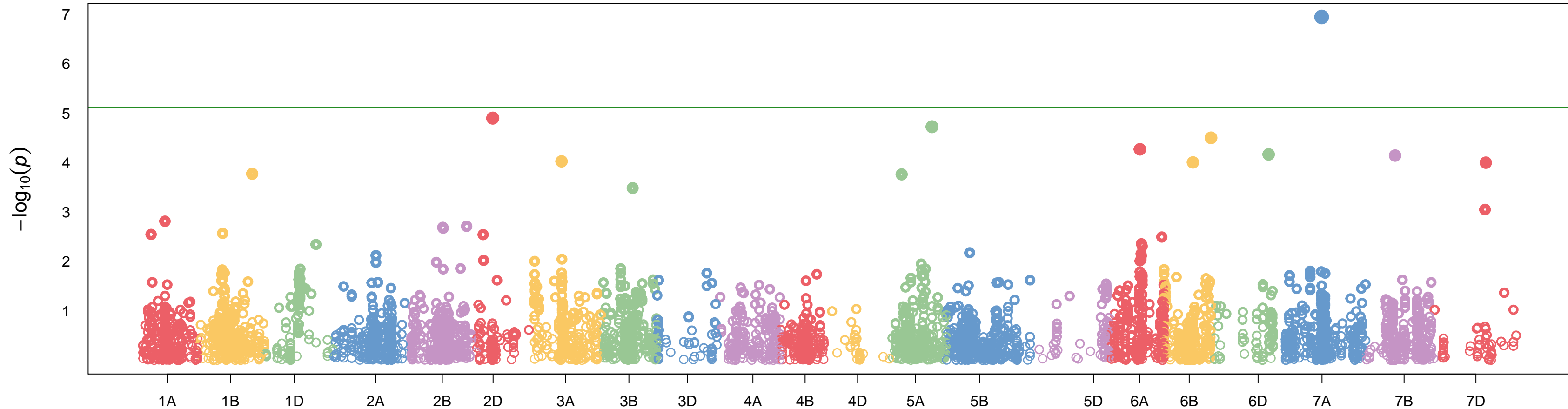

Supplement: Supplementary file 6 — Supplementary file6 (ZIP 14628 KB) [file 122_2023_4352_MOESM6_ESM.zip › SPL/5-Manhattan.Plot-SPL.pdf]

# FarmCPU.BLUP\_SPL

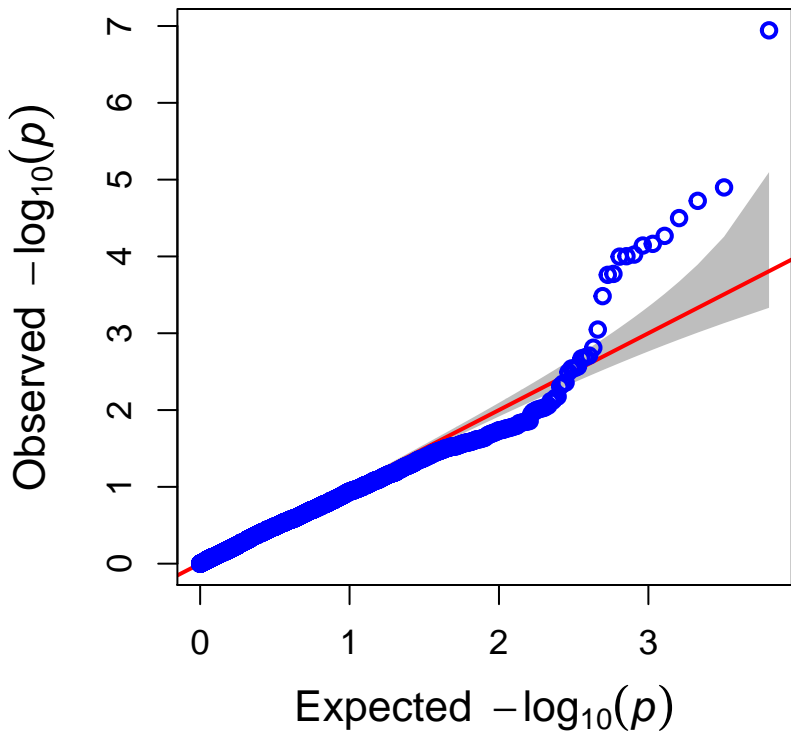

Supplement: Supplementary file 6 — Supplementary file6 (ZIP 14628 KB) [file 122_2023_4352_MOESM6_ESM.zip › SPL/5-QQ-Plot-SPL.pdf]

# FarmCPU.BLUP\_SPW

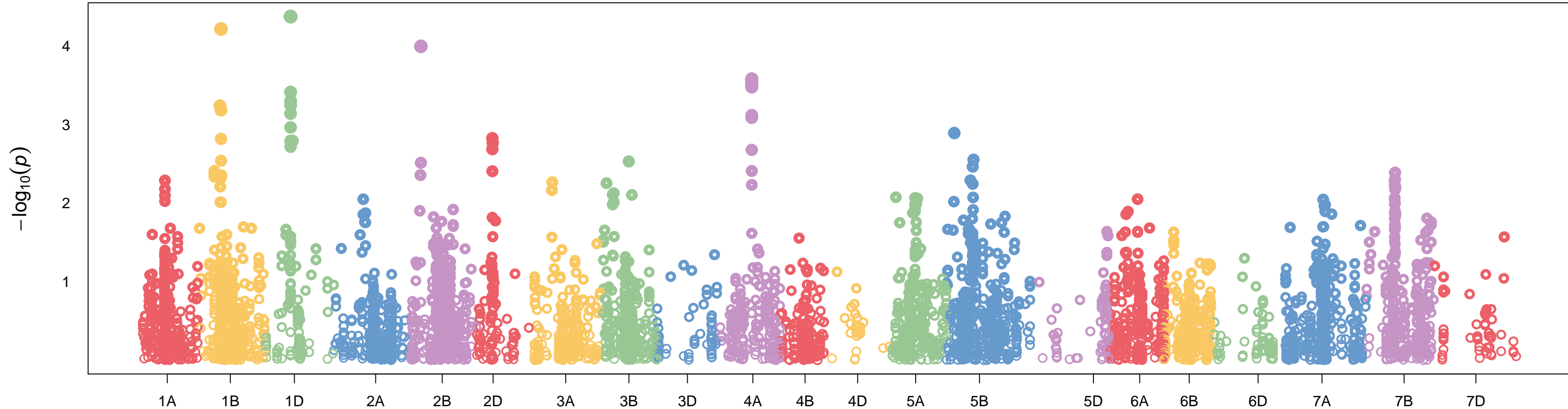

Supplement: Supplementary file 6 — Supplementary file6 (ZIP 14628 KB) [file 122_2023_4352_MOESM6_ESM.zip › SPW/1-Manhattan.Plot-SPW.pdf]

# FarmCPU.BLUP\_SPW

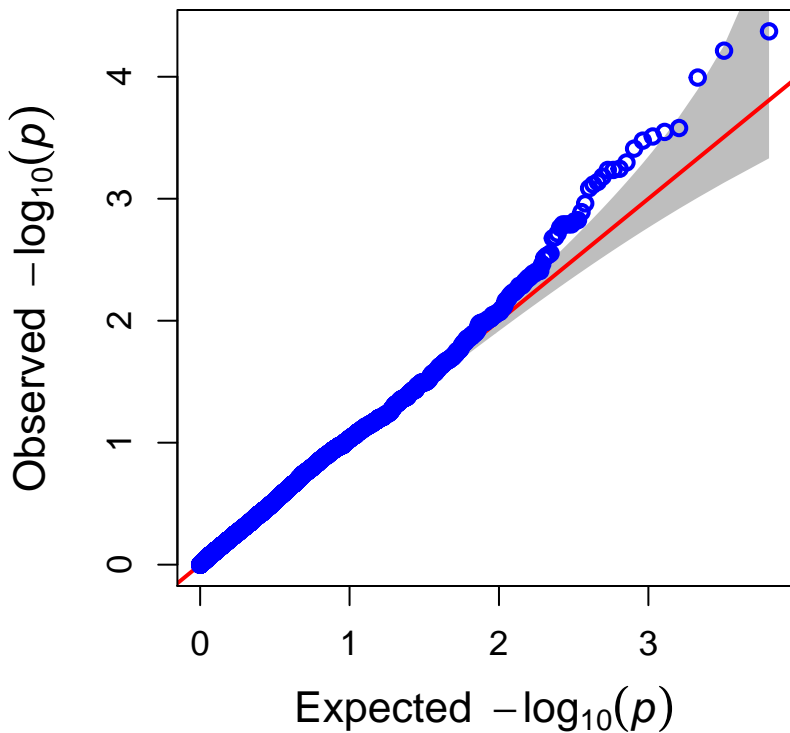

Supplement: Supplementary file 6 — Supplementary file6 (ZIP 14628 KB) [file 122_2023_4352_MOESM6_ESM.zip › SPW/1-QQ-Plot-SPW.pdf]

# FarmCPU.BLUP\_SPW

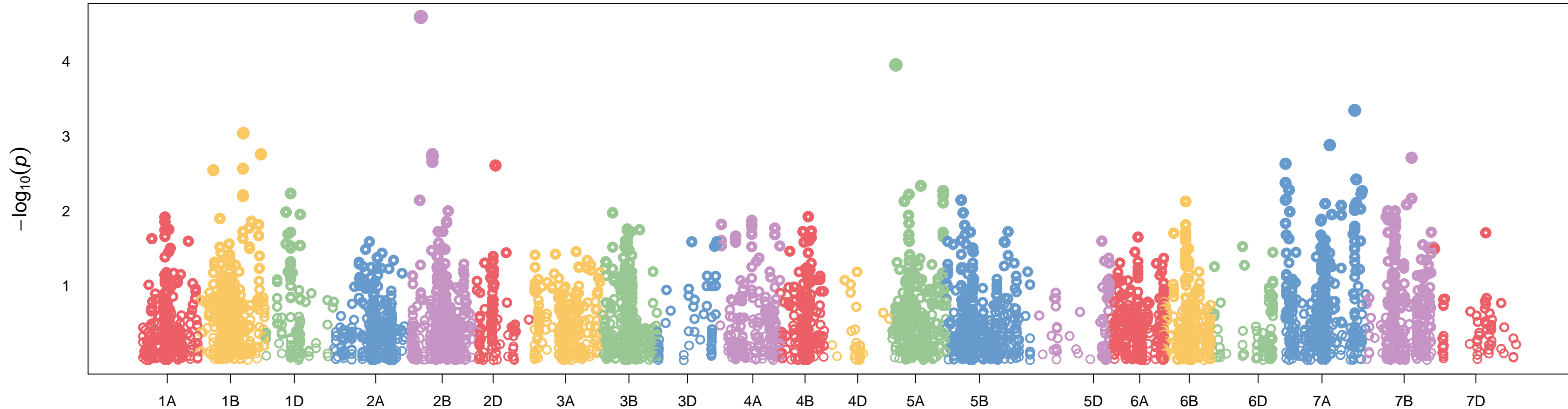

Supplement: Supplementary file 6 — Supplementary file6 (ZIP 14628 KB) [file 122_2023_4352_MOESM6_ESM.zip › SPW/2-Manhattan.Plot-SPW.pdf]

# FarmCPU.BLUP\_SPW

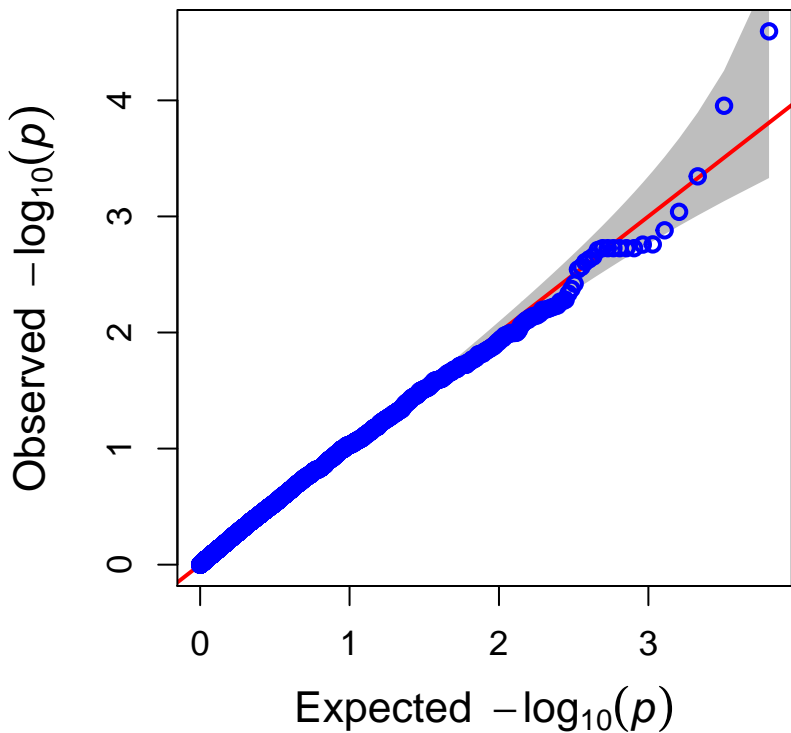

Supplement: Supplementary file 6 — Supplementary file6 (ZIP 14628 KB) [file 122_2023_4352_MOESM6_ESM.zip › SPW/2-QQ-Plot-SPW.pdf]

# FarmCPU.BLUP\_SPW

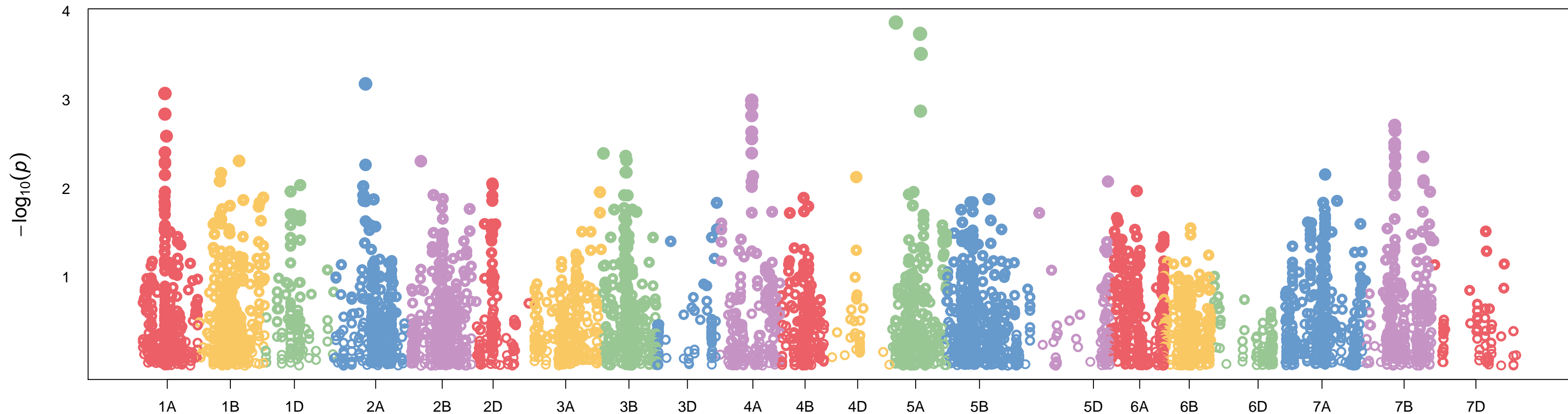

Supplement: Supplementary file 6 — Supplementary file6 (ZIP 14628 KB) [file 122_2023_4352_MOESM6_ESM.zip › SPW/3-Manhattan.Plot-SPW.pdf]

# FarmCPU.BLUP\_SPW

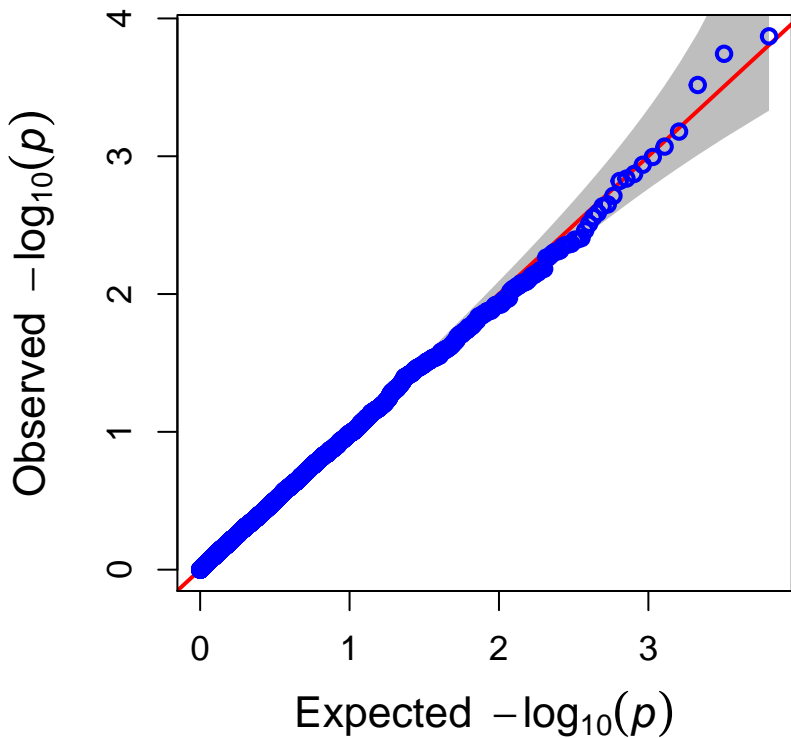

Supplement: Supplementary file 6 — Supplementary file6 (ZIP 14628 KB) [file 122_2023_4352_MOESM6_ESM.zip › SPW/3-QQ-Plot-SPW.pdf]

# FarmCPU.BLUP\_SPW

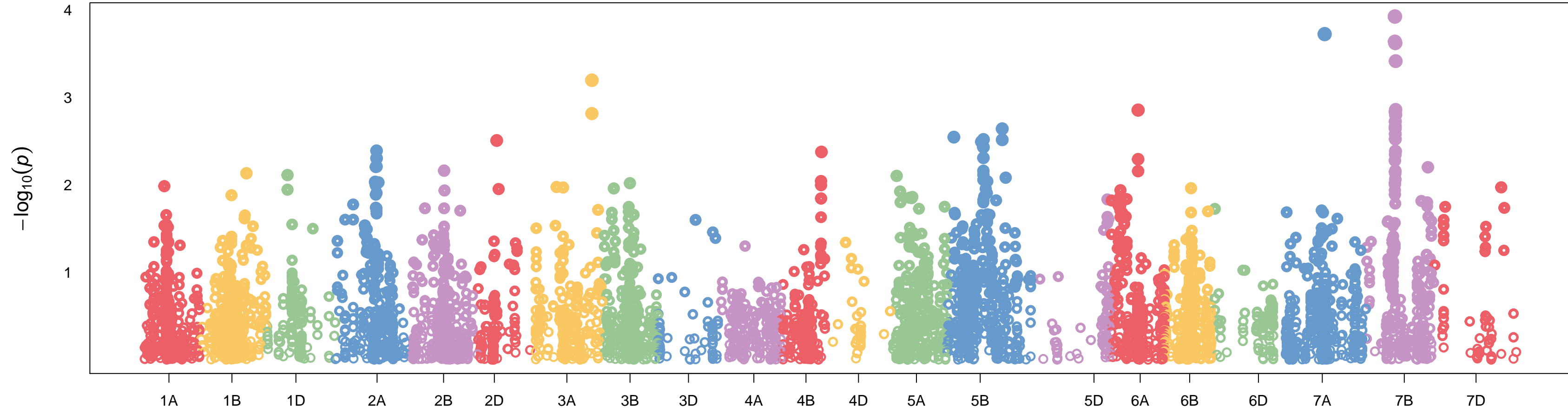

Supplement: Supplementary file 6 — Supplementary file6 (ZIP 14628 KB) [file 122_2023_4352_MOESM6_ESM.zip › SPW/4-Manhattan.Plot-SPW.pdf]

# FarmCPU.BLUP\_SPW

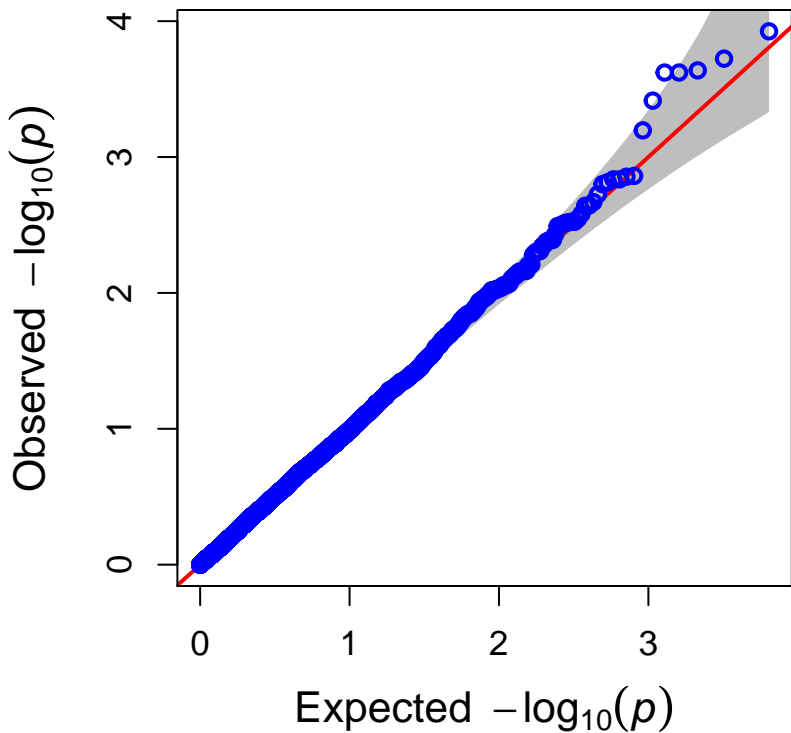

Supplement: Supplementary file 6 — Supplementary file6 (ZIP 14628 KB) [file 122_2023_4352_MOESM6_ESM.zip › SPW/4-QQ-Plot-SPW.pdf]

# FarmCPU.BLUP\_SPW

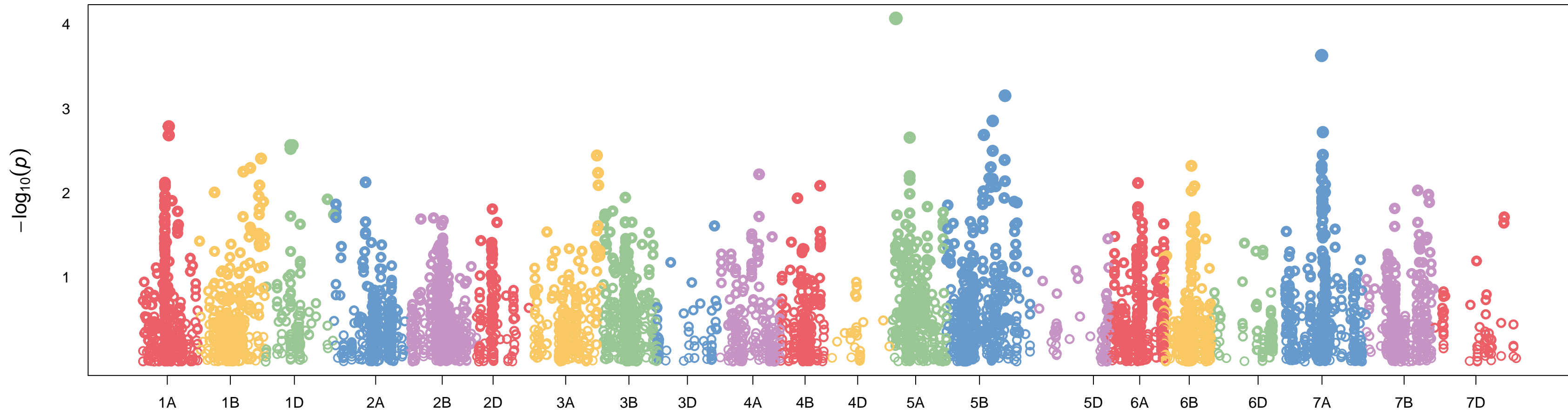

Supplement: Supplementary file 6 — Supplementary file6 (ZIP 14628 KB) [file 122_2023_4352_MOESM6_ESM.zip › SPW/5-Manhattan.Plot-SPW.pdf]

# FarmCPU.BLUP\_SPW

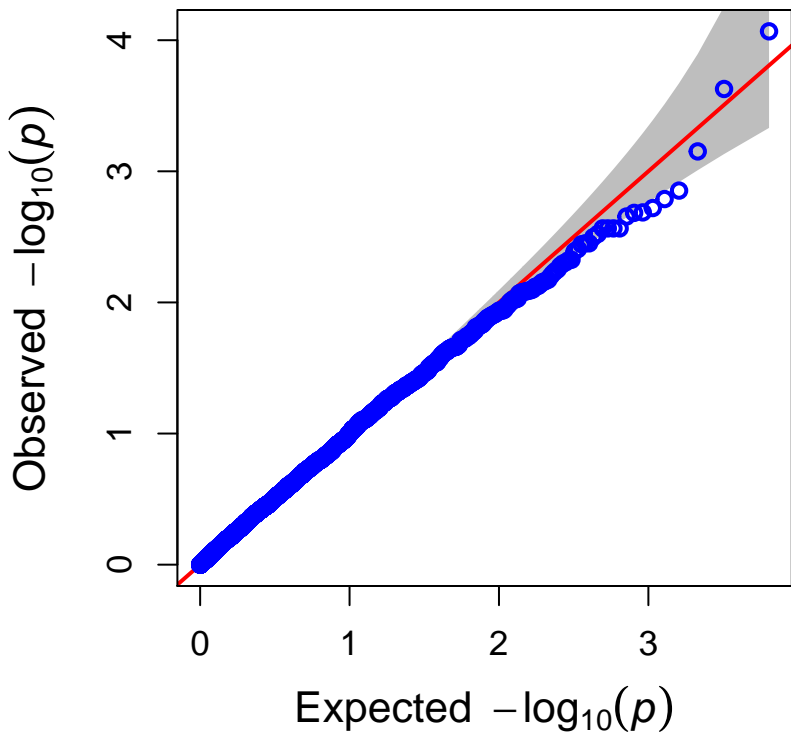

Supplement: Supplementary file 6 — Supplementary file6 (ZIP 14628 KB) [file 122_2023_4352_MOESM6_ESM.zip › SPW/5-QQ-Plot-SPW.pdf]
