# Supplementary material for: Haplotype-tagged SNPs improve genomic prediction accuracy for Fusarium head blight resistance and yield-related traits in wheat
Source: Theor Appl Genet. 2023 Apr 3;136(4):92. doi: 10.1007/s00122-023-04352-8 (PMC10068637; doi:10.1007/s00122-023-04352-8)
Supplement: Supplementary file 7 — Supplementary file7 (DOCX 1174 KB) [file 122_2023_4352_MOESM7_ESM.docx]

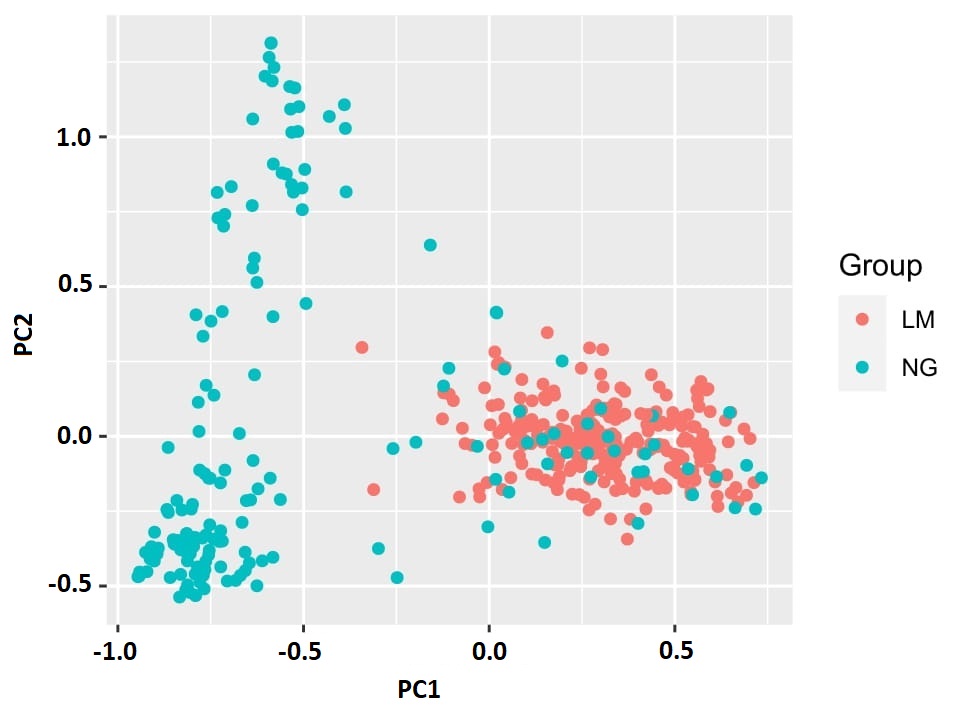


**Supplementary figure 1**. Principal component analysis of 419 genotypes comprising 272 advanced breeding lines from Lantmännen and 147 cultivars and landraces from the Nordgen. LM – Lantmännen breeding lines; NG – Nordgen cultivars and landraces.


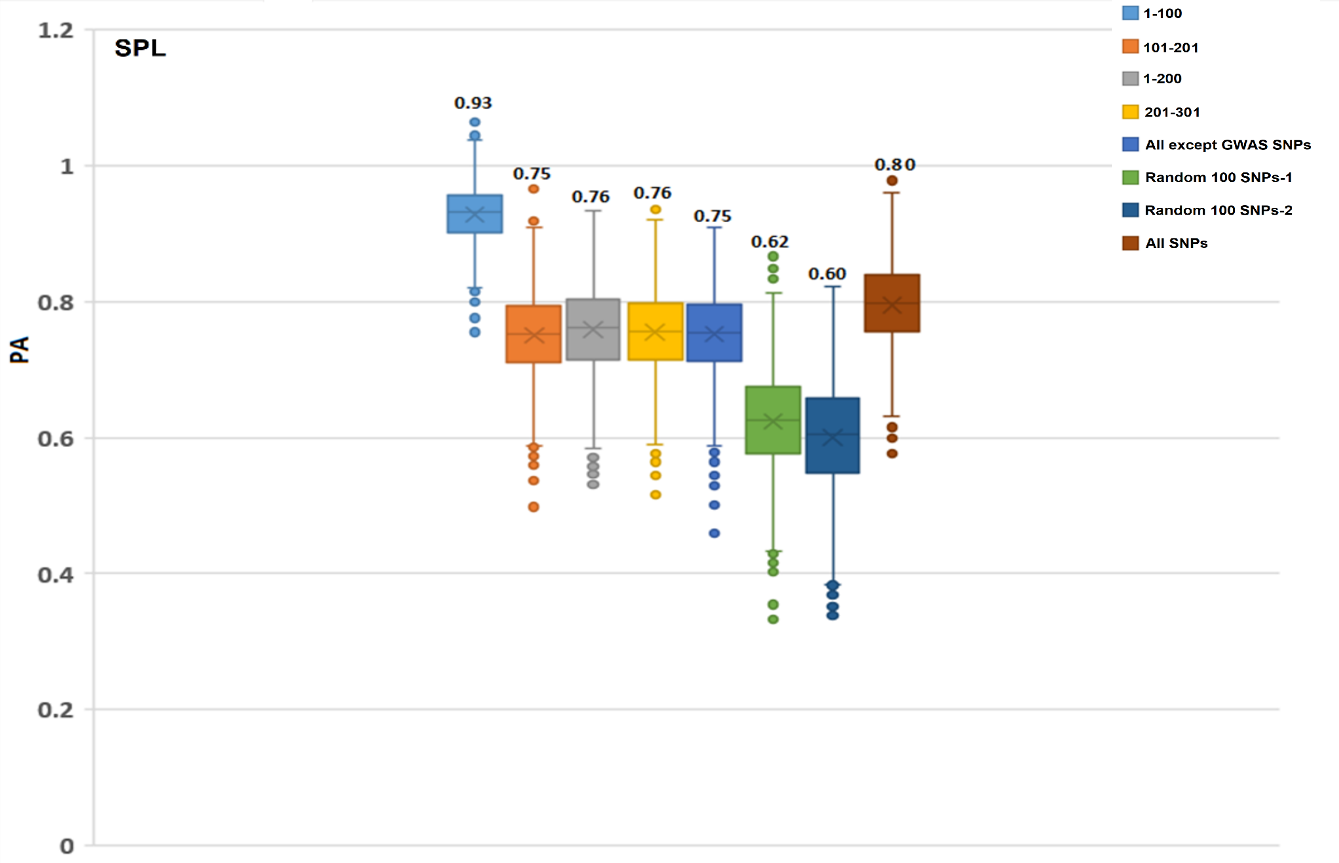

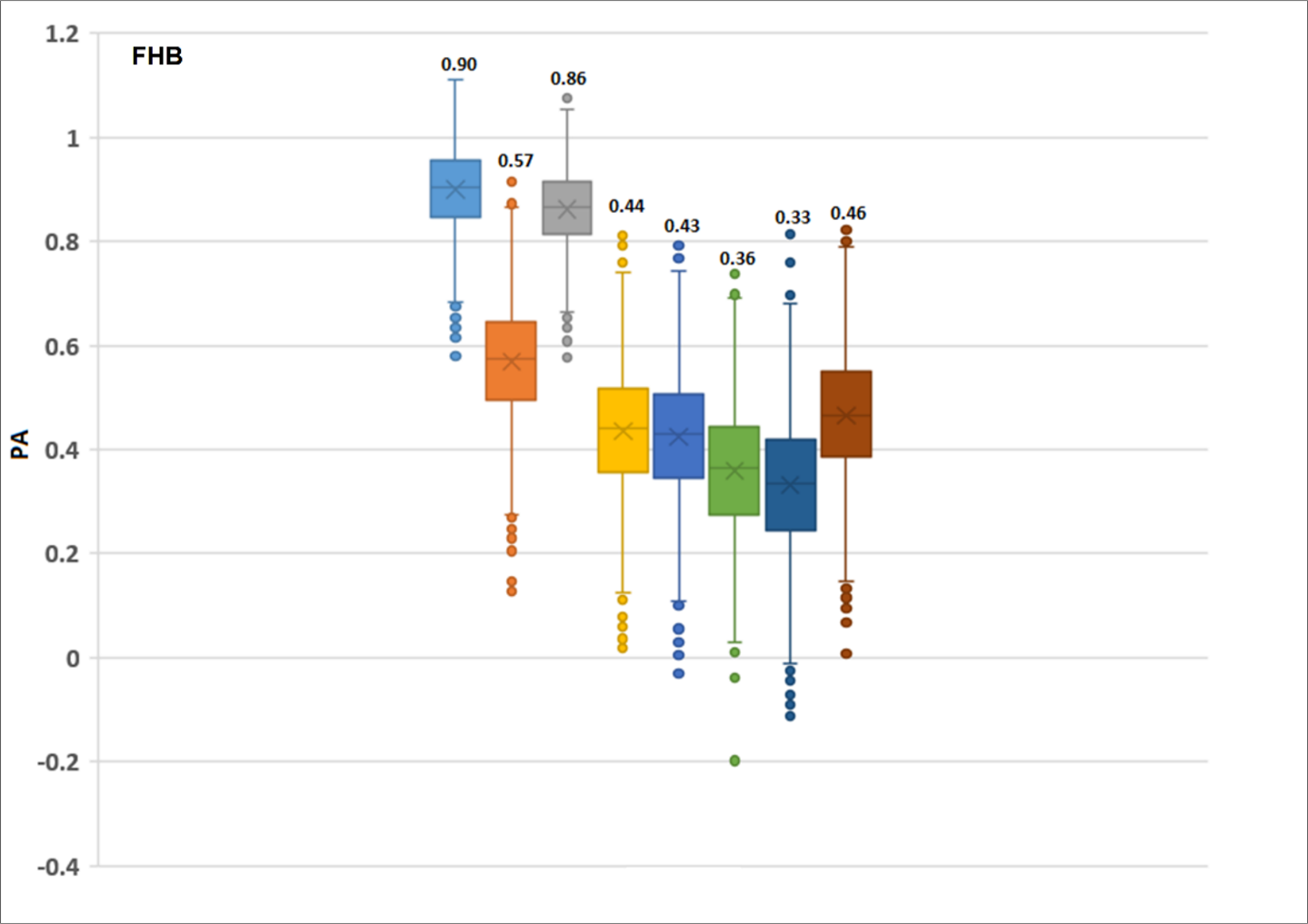


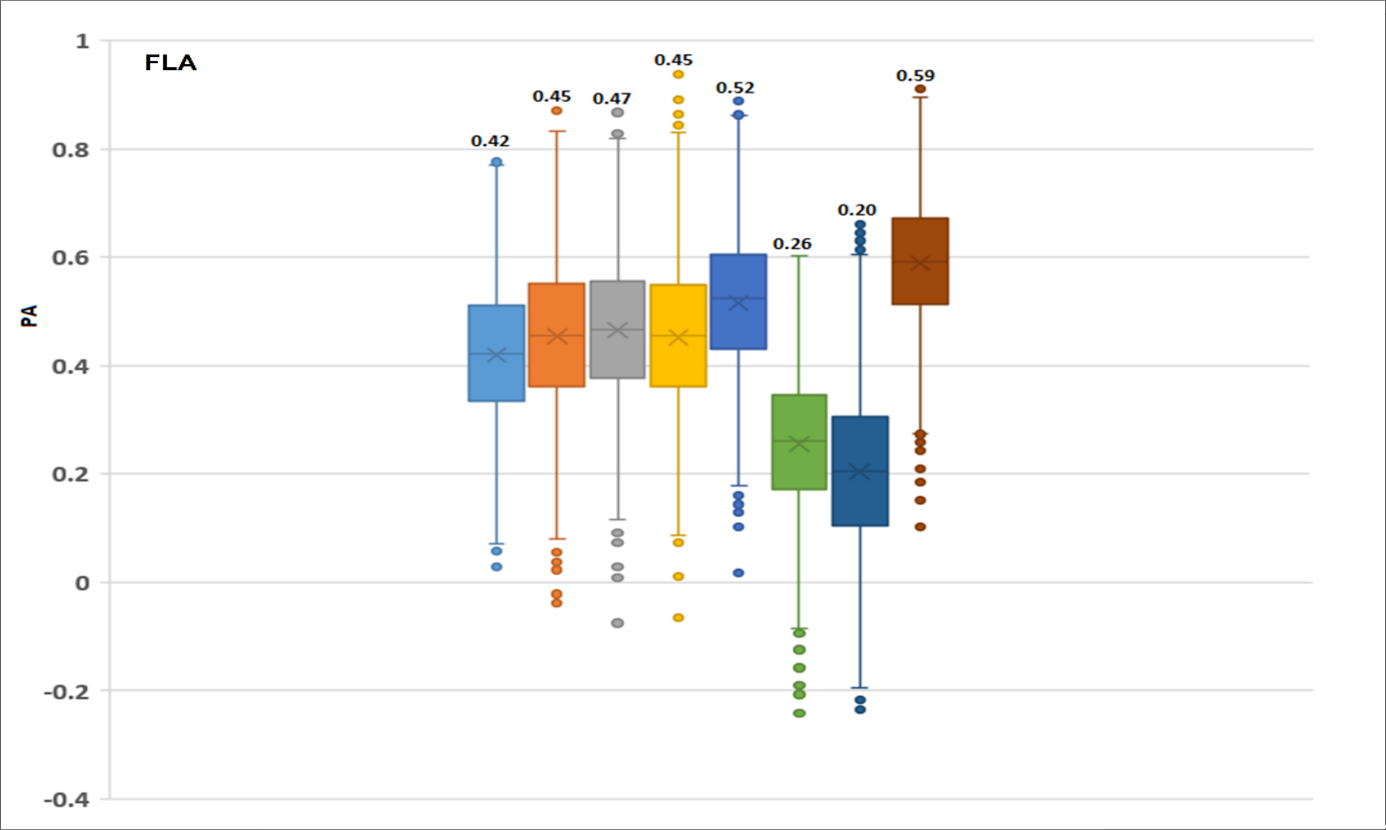

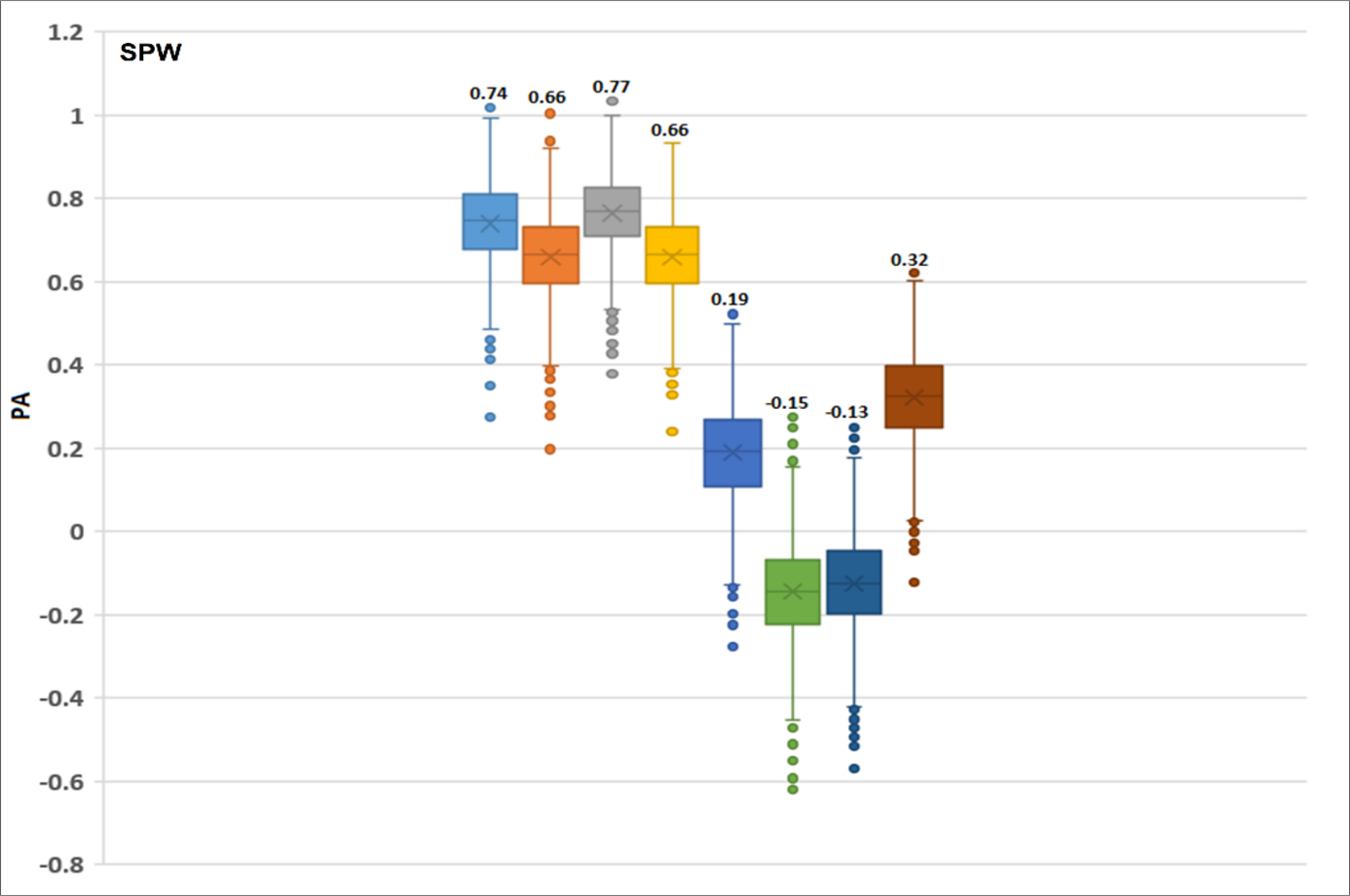


**Supplementary figure 2**. Genomic prediction accuracy with various sets of trait-linked SNP markers listed based on their significant effect. Numbers are the values of the genomic prediction accuracy averaged from 500 replications in RR-BLUP model. SPL, spike length; FHB, fusarium head blight resistance; SPW, spike width; FLA, flag leaf area.
